# EMbedding and Backscattered Scanning Electron Microscopy: A Detailed Protocol for the Whole-Specimen, High-Resolution Analysis of Cardiovascular Tissues

**DOI:** 10.3389/fcvm.2021.739549

**Published:** 2021-10-25

**Authors:** Rinat A. Mukhamadiyarov, Leo A. Bogdanov, Tatiana V. Glushkova, Daria K. Shishkova, Alexander E. Kostyunin, Vladislav A. Koshelev, Amin R. Shabaev, Alexey V. Frolov, Alexander N. Stasev, Anton A. Lyapin, Anton G. Kutikhin

**Affiliations:** Department of Experimental Medicine, Research Institute for Complex Issues of Cardiovascular Diseases, Kemerovo, Russia

**Keywords:** cardiovascular research, biocompatibility testing, calcification, mineral deposits, electron microscopy, sample preparation, epoxy resin, grinding and polishing

## Abstract

Currently, an ultrastructural analysis of cardiovascular tissues is significantly complicated. Routine histopathological examinations and immunohistochemical staining suffer from a relatively low resolution of light microscopy, whereas the fluorescence imaging of plaques and bioprosthetic heart valves yields considerable background noise from the convoluted extracellular matrix that often results in a low signal-to-noise ratio. Besides, the sectioning of calcified or stent-expanded blood vessels or mineralised heart valves leads to a critical loss of their integrity, demanding other methods to be developed. Here, we designed a conceptually novel approach that combines conventional formalin fixation, sequential incubation in heavy metal solutions (osmium tetroxide, uranyl acetate or lanthanides, and lead citrate), and the embedding of the whole specimen into epoxy resin to retain its integrity while accessing the region of interest by grinding and polishing. Upon carbon sputtering, the sample is visualised by means of backscattered scanning electron microscopy. The technique fully preserves calcified and stent-expanded tissues, permits a detailed analysis of vascular and valvular composition and architecture, enables discrimination between multiple cell types (including endothelial cells, vascular smooth muscle cells, fibroblasts, adipocytes, mast cells, foam cells, foreign-body giant cells, canonical macrophages, neutrophils, and lymphocytes) and microvascular identities (arterioles, venules, and capillaries), and gives a technical possibility for quantitating the number, area, and density of the blood vessels. Hence, we suggest that our approach is capable of providing a pathophysiological insight into cardiovascular disease development. The protocol does not require specific expertise and can be employed in virtually any laboratory that has a scanning electron microscope.

## Introduction

In spite of the variety of approaches for ultrastructural pathology, which generally include the preparation of formalin-fixed paraffin-embedded, snap-frozen, or fresh tissue specimens further stained with specific dyes or chromogen, fluorescent, or gold-labelled antibodies and visualised by light, epifluorescence, confocal, or electron microscopy (1–3), the processing and imaging of calcified or stent-expanded cardiovascular tissue remain poor (4). The density of mineral deposits and metal implants significantly differs from that of the bulk tissue and, hence, tissue integrity is heavily disrupted through the conventional sectioning procedure. Yet, a proper investigation of atherosclerotic plaques, dysfunctional heart valves, and failed bioprosthetic heart valves all frequently undergoing pathological mineralisation ultimately requires retained tissue architecture and the biocompatibility testing of the metal alloys for stent manufacturing (5, 6).

Another major issue in cardiovascular pathology is that the high-precision and high-throughput analysis of microcirculation demands a magnification exceeding values obtainable by means of light microscopy (LM, 400-fold) and an image acquisition rate superior to confocal microscopy (which is commonly limited to 630-fold magnification, being additionally confounded by antigen expression features and background staining) (7, 8). Transmission electron microscopy provides a perfect magnification range, but the sample preparation is technically challenging, is inevitably associated with a tremendous reduction of the sample amount, and does not permit the representative serial sectioning of the vessels (9). Hence, the existing techniques for the visualisation of microcirculation have considerable shortcomings and also involve sectioning prior to the staining, precluding an analysis of the vascular networks associated with extraskeletal mineral deposits.

An established standard for the evaluation of small-calibre vessels includes immunostaining for endothelial marker CD31 in combination with nuclear counterstaining (10). However, the geometry of microvessels is susceptible to sectioning and staining and often becomes irreversibly altered during these procedures (in particular in calcified or stent-expanded tissues) that complicates the evaluation of microcirculation. Immunostaining for vascular smooth muscle cell markers, such as the smooth muscle myosin heavy chain, alpha smooth muscle actin, or smoothelin, better delineates microvessel contours but does not detect the capillaries, which solely consist of endothelial cells. Therefore, despite excellent specificity and compatibility with recent tools for automated image analysis, immunostaining lacks sensitivity in discriminating microvasculature from surrounding tissue.

Here we developed and validated a conceptually novel histological approach that couples whole-specimen formalin fixation with heavy metal staining (i.e., incubation in osmium tetroxide, uranyl acetate, and lead citrate solutions), epoxy resin embedding, grinding, and polishing of epoxy resin blocks, and backscattered scanning electron microscopy (BSEM). As this technique fully retains the integrity of calcified or stent-expanded tissues and combines high-magnification visualisation, rapid image acquisition, and the possibility to perform elemental analysis, we suggest it as an optimal solution for cardiovascular research, especially for studies on calcification and microcirculation.

### Development of the Protocol

To design this protocol, we combined a classical tissue fixation in neutral phosphate buffered formalin—which, in this setting, is not restricted to 24 h as in the case with immunohistochemistry—with post-fixation and long-term staining with ascending concentrations of osmium tetroxide. After washing and dehydration, samples are counterstained in alcoholic uranyl acetate (or its substitutes such as lanthanides if desired), impregnated and embedded into epoxy resin, and then grinded and polished to retrieve the sample and flatten its surface for electron microscopy. Visualisation is performed utilising BSEM upon counterstaining with lead citrate and carbon sputtering. The protocol provides an opportunity to investigate the entire and intact tissue sample, conduct layer-by-layer examination by serial grinding, and acquire high-quality images of tissue architecture, extracellular matrix, and cells at a magnification from 40- to 5,000-fold. For the investigation of mineral deposits or metal implants, visual inspection can be reinforced by an elemental analysis. Employing a proposed technique, we carried out an ultrastructural analysis of atherosclerotic plaques as compared with failing native and bioprosthetic heart valves, posing the integrity of the extracellular matrix as a pivotal factor for the prevention of structural valve deterioration (11).

### Applications of the Method

The histological interrogation of blood vessels and heart valves and their polymer and bioprosthetic substitutes is frequently complicated by an extensive calcification, which leads to the loss of tissue integrity during the sectioning. Our approach avoids this drawback as it implies processing of the entire tissue explant through all sample preparation stages. Moreover, it adds the ability to analyse the chemical composition of the minerals or implants. Furthermore, the magnification of BSEM is optimal for the analysis of vascular architecture, allowing the clear visualisation of the endothelium, tunica intima, vascular smooth muscle cell layers, elastic fibres, collagen meshwork, *vasa vasorum*, plaque neovessels, immune cell clusters, peripheral nerves, and perivascular adipose tissue. Alterations detectable by our approach include but are not limited to extraskeletal calcification, lipid retention and foam cell formation, intraplaque or intravalvular haemorrhages, elastic lamina degradation, and neutrophil adhesion/migration. By optimising the staining and dehydration protocol, BSEM distinguishes endothelial cells, vascular smooth muscle cells, fibroblasts, mast cells, neutrophils, macrophages (including foam cells and foreign-body giant cells), lymphocytes, and perivascular adipocytes. The applicability of our technique for vascular pathology tasks has been confirmed on a sample of coronary artery bypass graft surgery conduits (saphenous veins and internal mammary arteries), demonstrating an association of increased *vasa vasorum* density with pre-implantation stenosis (12). An independent validation sample included balloon-injured rat aortas, which indicated the correlation of *vasa vasorum*, a surrogate vascular inflammation marker, with immune cell clusters also associated with neointima formation (13). Finally, we have been able to show the relation of plaque neovessels with calcium deposition, further reporting the positive role of the microvessels around the mineral deposits and the negative role of total plaque vascularisation (14).

### Experimental Design

This protocol provides a step-by-step guide to prepare the blood vessels, heart valves, and their polymer and bioprosthetic substitutes for the analysis of their gross and microscopic anatomy by BSEM. It can be divided into the following stages: (i) fixation, staining, and preparation for epoxy resin embedding; (ii) embedding into epoxy resin and preparation of the sample for BSEM; (iii) BSEM visualisation and analysis. The protocol, although being developed for studying cardiovascular pathology, is not restricted to this field and can be modified at any stage to optimise the results with respect to specific organs and tissues. The procedure described in the study has been optimised for atherosclerotic plaques, native and bioprosthetic heart valves, coronary artery bypass graft surgery conduits (autologous internal mammary arteries and saphenous veins), ovine carotid arteries (including those with implanted tissue-engineered vascular grafts), and rat aortas. We expect the performance of the method would be similar if applied to any other human or animal organ or tissue.

#### Fixation, Staining, and Preparation for Epoxy Resin Embedding (Steps 1–18)

This part of the protocol includes the standard fixation of the tissue in neutral phosphate buffered formalin immediately upon its collection, postfixation, and staining in ascending concentrations of osmium tetroxide, dehydration steps combined with the counterstaining in alcoholic uranyl acetate or lanthanides, and impregnation into epoxy resin. Although the modification and even substitution of some steps here are possible without affecting the result, we recommend adhering to the protocol in this part as it employs the basic principles of histology.

#### Embedding into Epoxy Resin and Preparation of the Sample for BSEM (Steps 19–24)

Here, we describe a critical step (sample orientation and epoxy resin embedding) of further sample retrieval by grinding and polishing and the final preparations that ensure high-quality BSEM (lead citrate counterstaining and carbon sputter coating). Serial grinding can be employed for the sequential scanning of the sample at ascending tissue depth. Various types of epoxy resin may be used here depending on the desired penetration into the tissue and subsequent modes of analysis. We recommend using Araldite 502 (in preference to Araldite-EMbed 812, Embed-812, and Spurr low-viscosity resin), a widely utilised embedding resin for embedding, which provides advantages that include rapid penetration, good contrasting properties, easy grinding, and stability under the electron beam. Different variants of lead citrate for counterstaining are available, yet we refer to Reynolds' formulation. Monostaining with lanthanides facilitates the further elemental analysis of mineral deposits, if applicable, in comparison with uranyl acetate/lead citrate treatment.

#### Backscattered Scanning Electron Microscopy Visualisation (Step 25)

Upon sample preparation, an ultrastructural analysis can be conducted by means of BSEM, which provides a high-resolution image from ×40 to ×5,000 magnification. This imaging modality is similar to transmission electron microscopy (TEM) in terms of visualisation patterns, facilitating the integration of EM-BSEM into the existing electron microscopy techniques applied in histopathology. Furthermore, BSEM is fully compatible with elemental analysis (e.g., energy-dispersive x-ray spectroscopy) and automated machine learning algorithms for post-acquisition image analysis.

### Expertise Needed to Implement the Protocol

The implementation of the protocol does not require any specific expertise, and all steps can be performed by a competent graduate student or postdoctoral researcher without the need to involve a core facility or the use of a specific protective equipment. Yet, a grinding and polishing machine, a vacuum sputter coater, and a scanning electron microscope equipped with a backscattered electron detector are mandatory for the experiments utilising this protocol. The workflow is reminiscent of a routine histological sample preparation and includes the sequential incubation of the sample(s) in various chemical solutions, the orientation of the specimen in the embedding mould, grinding and polishing upon epoxy resin polymerisation, counterstaining followed by a sputter coating, and BSEM. The acquisition of relevant and high-quality images and their interpretation and semi-quantitative analysis require expertise and, ideally, experience in cardiovascular pathology (or a respective pathology field if studying other tissues). The development of neural networks for the automated analysis of acquired images would demand skills in data science.

## Materials and Equipment

### Biological Materials

1. Although the approach has been optimised for cardiovascular tissues, it can be applied to virtually any tissue. **CAUTION:** For all materials collected from live vertebrates or higher invertebrates, all experiments must be performed in accordance with relevant guidelines and regulations (e.g., European Convention for the Protection of Vertebrate Animals used for Experimental and Other Scientific Purposes, Strasbourg, 1986). For all materials obtained from human subjects, their collection must conform to the latest revision of Declaration of Helsinki (2013), and written informed consent to participate in the study must be obtained from all subjects.

### Reagents

1. Ice-cold 1X phosphate-buffered saline (e.g., P4417, Sigma-Aldrich; St. Louis, MO, USA, dissolve five tablets in 1,000 ml of double-distilled water) or a physiological saline solution [0.9% (wt/vol) NaCl, can be prepared by dissolving 9 g of NaCl (e.g., 31434, Sigma-Aldrich) in 1,000 ml of double-distilled or deionised water] as a transportation medium for the tissue samples. Can be stored at 4°C for 3 months.
  **CRITICAL:** All reagents mentioned below must not be of a grade less than the ACS grade (≥99% purity) to obtain high-quality images.
2. Ice-cold 10% (vol/vol) neutral phosphate-buffered formalin (e.g., HT501128, Sigma-Aldrich) for the sample fixation by: (1) protein–protein and protein–nucleic acid cross-linking *via* amino or imino groups including those of adenine and cytosine (15–18); (2) reacting with the ethylenic double bonds of unsaturated lipids (19, 20). Can be prepared by dissolving 4 g of anhydrous monobasic sodium phosphate (NaH_2_PO_4_, e.g., S0751, Sigma-Aldrich) and 6.5 g of anhydrous dibasic sodium phosphate (Na_2_HPO_4_, e.g., S0876, Sigma-Aldrich) in 900 ml of double-distilled or deionised water and adding 100 ml of 37% (wt/vol) formaldehyde (e.g., 15680, Electron Microscopy Sciences; Hatfield, PA, USA or 252549, Sigma-Aldrich). Can be stored at 4°C for 1 year.
  **CAUTION:** Fatal if swallowed, causes severe skin and eye irritation, may cause respiratory irritation, allergy or asthma symptoms, or breathing difficulties if inhaled. Wear protective clothing (gloves and face protection), do not breathe dust/fume/gas/mist/vapours/spray, wash gloves and hands thoroughly after handling, use only in a well-ventilated area such as a fume hood.
3. One percent (wt/vol) phosphate-buffered osmium tetroxide (OsO_4_, e.g., 19100, Electron Microscopy Sciences) for the sample post-fixation by: (1) the polymerisation of unsaturated lipids through the cross-linking of ethylenic double bonds (21, 22); (2) protein–protein cross-linking *via* amino/imino groups (22, 23); (3) protein–lipid cross-linking by an unrecognised mechanism (23). Prepare by dissolving 1 g of osmium tetroxide in 100 ml of 0.1 M phosphate buffer [pH 7.4, can be prepared by: (1) dissolving 14.2 g of anhydrous Na_2_HPO_4_ in 1 L of double-distilled or deionised water; (2) dissolving 12 g of anhydrous NaH_2_PO_4_ in 1 L of double-distilled or deionised water; (3) mixing 405 ml of a Na_2_HPO_4_ solution with 95 ml of a NaH_2_PO_4_ solution, can be stored at 4°C for 1 year]. Can be stored tightly wrapped into a sealing film (e.g., Parafilm M, P7793, Sigma-Aldrich) at 4°C for 3 months. Protect from light.
  **CAUTION:** Fatal if swallowed, in contact with skin, or inhaled and causes severe skin burns and eye damage. Wear protective clothing (gloves and face protection), do not breathe dust or mists, wash gloves and hands thoroughly after handling, use only in a well-ventilated area such as a fume hood, and strictly use glass vials (e.g., 23188, Supelco) for incubating the specimens, as glass is a chemically inert material that does not react with osmium tetroxide, in contrast to plastic.
4. Two percent (wt/vol) aqueous osmium tetroxide (OsO_4_, e.g., 19100, Electron Microscopy Sciences) to better stain proteins and lipids. Prepare by dissolving 1 g of osmium tetroxide in 50 mL double-distilled or deionised water. Can be stored tightly wrapped into a sealing film at 4°C for 3 months. Protect from light.
  **CAUTION:** Fatal if swallowed, in contact with skin, or inhaled and causes severe skin burns and eye damage. Wear protective clothing (gloves and face protection), do not breathe dust or mists, wash gloves and hands thoroughly after handling, use only in a well-ventilated area such as a fume hood, and strictly use glass vials for incubating the specimens, as glass is a chemically inert material that does not react with osmium tetroxide, in contrast to plastic.
5. Absolute ethanol (CH_3_CH_2_OH, 200 proof, e.g., 15058, Electron Microscopy Sciences) further diluted to 95 (vol/vol), 80 (vol/vol), 70 (vol/vol), 60 (vol/vol), and 50% (vol/vol) ethanol in double-distilled or deionised water. Such graded ethanol series is used for tissue dehydration and mild delipidation. Store at room temperature (RT).
  **CAUTION:** Highly flammable liquid and vapour, harmful if swallowed or in contact with skin, and causes skin and eye irritation. Keep away from heat/sparks/open flames/hot surfaces, do not smoke around reagent, use explosion-proof electrical/ventilating/lighting/equipment, use only non-sparking tools, take precautionary measures against static discharge, wear protective clothing (gloves and face protection), and wash gloves and hands thoroughly after handling.
6. Uranyl acetate (C_4_H_6_O_6_U, e.g., 22400, Electron Microscopy Sciences) further diluted to 2% (wt/vol) in 95% (vol/vol) ethanol (e.g., add 25 ml of 95% ethanol to 0.5 g of uranyl acetate). Uranyl acetate binds to sialic acid carboxyl groups of glycoproteins and gangliosides abundant in cell membranes, to amino groups of proteins, and to phosphate groups of nucleic acids (24), thereby contrasting nuclei and ribosomes. Must be prepared *ex tempore* and left for 4 h to settle. Protect from air and light.
  **CAUTION:** Highly flammable liquid and vapour, toxic if swallowed, inhaled or in contact with skin, causes serious skin and eye irritation, and suspected of causing genetic defects and damaging fertility or the unborn child. Keep away from heat/sparks/open flames/hot surfaces, do not smoke around reagent, use explosion-proof electrical/ventilating/lighting/equipment, use only non-sparking tools, take precautionary measures against static discharge, do not breathe dust/fume/gas/mist/vapours/spray, wear protective clothing (gloves and face protection), and wash gloves and hands thoroughly after handling.
  **ALTERNATIVE:** Use undiluted UranyLess, a proprietary mix of lanthanides (e.g., 22409, Electron Microscopy Sciences; Hatfield, PA, USA) binding to calcium, phosphates, and phospholipids and to amino groups of proteins and therefore staining cell membranes, organelles, and nuclei (25–30). Compared with uranyl acetate, UranyLess is a harmless reagent and generally shows similar efficiency to a contrasting agent. Store at RT for 1 year. Protect from light.
7. 2-propanol {[(CH_3_)_2_CHOH], e.g., 190764, Sigma-Aldrich}, a degreasing and dehydrating agent. More potent degreasing agent than ethanol. Store at RT.
  **CAUTION:** Highly flammable liquid and vapour, harmful if swallowed or in contact with skin, causes skin and eye irritation, a d may cause drowsiness or dizziness. Keep away from heat/sparks/open flames/hot surfaces, do not smoke around the reagent, use explosion-proof electrical/ventilating/lighting/equipment, use only non-sparking tools, take precautionary measures against static discharge, wear protective clothing (gloves and face protection), and wash gloves and hands thoroughly after handling.
8. Acetone [(CH_3_)_2_CO], e.g., 179124, Sigma-Aldrich, a more potent dehydrating and degreasing agent as compared with ethanol and 2-propanol. Miscible with epoxy resin and less toxic as compared with propylene oxide. Store at RT.
  **CAUTION:** Highly flammable liquid and vapour, toxic if swallowed or inhaled, causes skin and eye irritation, and may cause drowsiness or dizziness. Keep away from heat/sparks/open flames/hot surfaces, do not smoke around reagent, use explosion-proof electrical/ventilating/lighting/equipment, use only non-sparking tools, take precautionary measures against static discharge, wear protective clothing (gloves and face protection), wash gloves and hands thoroughly after handling, and use only in a well-ventilated area such as a fume hood.
9. Epoxy resin: Araldite 502 (e.g., 13900, Electron Microscopy Sciences), Araldite-EMbed 812 (e.g., 13940, Electron Microscopy Sciences), EMbed-812 (e.g., 14120 or 14121, Electron Microscopy Sciences), or Spurr low-viscosity resin (e.g., 14300, Electron Microscopy Sciences). We recommend using Araldite 502 as an option of choice, while EMbed-812 or Spurr low-viscosity resin may show better results when applied to bone tissues or samples with a high proportion of calcified tissue. Must be prepared *ex tempore*. To prepare Araldite 502, mix 20 ml of Araldite 502, 22 ml of dodecenyl succinic anhydride (a hardener), and 0.8 ml of DMP-30 (an accelerator). To prepare Araldite-EMbed 812, mix 25 ml of EMbed-812, 15 ml of Araldite 502, 55 ml of dodecenyl succinic anhydride, and 1.9 ml of DMP-30. To prepare EMbed-812, mix 20 ml of EMbed-812, 22/16/9 ml of dodecenyl succinic anhydride (for soft/medium/hard blocks, respectively), 5/8/12 ml of nadic methyl anhydride (another hardener, for soft/medium/hard blocks, respectively), and 0.9, 0.85, and 0.80 ml of DMP-30 (an accelerator, for soft/medium/hard blocks, respectively). To prepare the Spurr low-viscosity resin, mix 18 ml of ERL 4221, 14 ml of DER 736, 48 ml of non-enyl succinic anhydride (a hardener), and 0.6 ml of dimethylaminoethanol (an accelerator). Retain the abovementioned proportions when preparing larger epoxy resin amounts.
  **CAUTION:** Harmful if swallowed, in contact with skin, or inhaled, causes serious skin and eye irritation, and may cause respiratory irritation and allergic skin reaction. Do not breathe dust/fume/gas/mist/vapours/spray, wear protective clothing (gloves and face protection), wash gloves and hands thoroughly after handling, and use only in a well-ventilated area such as a fume hood. Additionally, dimethylaminoethanol (an accelerator for Spurr low-viscosity resin) is a flammable liquid and vapour and causes severe skin burns and eye damage. When working with this reagent, keep away from heat/sparks/open flames/hot surfaces, do not smoke around reagent, use explosion-proof electrical/ventilating/lighting/equipment, use only non-sparking tools, and take precautionary measures against static discharge.
10. Silicone oil (e.g., 40300076, Struers; Sarasota, FL, USA) to facilitate the removing of epoxy resin blocks from the moulds upon the embedding.
11. Graded (9, 6, and 3 μm in diameter) diamond spray series for the polishing of epoxy resin blocks (e.g., DP-Spray M, 40600154, 40600153, and 40600152, Struers).
12. Water-based cooling and lubricating liquid for the diamond polishing of epoxy resin blocks (e.g., DP-Lubricant Green, 40700024, Struers).
13. Lead citrate trihydrate [Pb(C_6_H_5_O_7_)_2_·3H_2_O, e.g., 17800, Electron Microscopy Sciences] to further contrast lipids and proteins as it interacts with both osmium tetroxide and uranyl acetate and also binds to cysteine, ortho- and pyrophosphate groups, and glycogen (31). Alternatively, it can be prepared by adding 1.33 g of lead nitrate (e.g., 17900, Electron Microscopy Sciences), 1.76 g of sodium citrate (e.g., 21140, Electron Microscopy Sciences), and 30 ml of fresh double-distilled or deionised water into a 50 ml conical tube (e.g., 91050, Techno Plastic Products; Trasadingen, Switzerland). Shake vigorously for 1 min and intermittently for 30 min. Then, add 8 ml of 1 N NaOH to the mixture and swirl. Again, add fresh double-distilled or deionised water to reach a 50-ml final volume. Shake vigorously for 1 min and cheque the pH (must be 11.9–12.1). Adjust the pH with 1 N NaOH if needed while stirring the solution. Pass the solution through a 0.2-μm pore size regenerated cellulose syringe philtre (e.g., 431222, Corning; Corning, NY, USA). It can be stored tightly wrapped into a sealing film at 4°C for 6 months. Protect from air and light.
  **CAUTION:** Toxic if swallowed or inhaled and may damage fertility or the unborn child. Do not breathe dust/fume/gas/mist/vapours/spray, wear protective clothing (gloves and face protection), wash gloves and hands thoroughly after handling, and use only in a well-ventilated area such as a fume hood.

### Consumables

1. Polypropylene mounting cups (e.g., FixiForm, 40300085, Struers) acting as a mould for epoxy resin embedding.
2. Silicon carbide foil (200 mm in diameter) with poly- (ethylene terephthalate) foil backing for the wet grinding of materials (HV 30–800); grit 800 (e.g., 40400206, Struers), 1,000 (e.g., 40400207, Struers), and 1,200 (e.g., 40400208, Struers) for reaching the sample surface. These must be attached to an adapter for magnetic fixation on an aluminium disc of the grinding and polishing machine (e.g., MD-Gekko, 200 mm in diameter, 49900047, Struers) or used with a self-adhesive foil (e.g., Gekko PSA, 200 mm in diameter, e.g., 49900053, Struers), which is to be glued on an aluminium disc.
3. Wool woven polishing cloth discs to use with 6- or 3-μm diameter monocrystalline diamonds for flattening the surface of the epoxy resin block (e.g., M300, NX-MET; Échirolles, France).
4. Carbon thread for sputter coating (e.g., 16771511116, Leica Microsystems; Wetzlar, Germany).
5. Glass Petri dish with lid for lead citrate counterstaining (e.g., 100 mm in diameter and 20 mm in height, BR455743, Sigma-Aldrich).
6. Laboratory glass bottles, 100–1,000 ml (e.g., Duran graduated laboratory bottles, Z232076, Z232084, Z232092, and Z232106), and their respective caps (GL 32 and GL 45 neck thread, Z232351 and Z153958) for preparing the phosphate-buffered and physiological saline, neutral phosphate-buffered formalin, osmium tetroxide, uranyl acetate, and ethanol dilutions.
7. Laboratory glass measuring cylinders, 10, 50, and 1,000 ml (e.g., BR31708, BR31728, and BR31762, Sigma-Aldrich), for measuring chemical solutions.
8. Laboratory glass beakers, 10 ml (e.g., Z231797, Sigma-Aldrich), for washing the specimens.
9. Laboratory glass vials (with caps), 40 ml (e.g., 23188, Supelco), for incubating the specimens. Ensure that the diameter of your vial fits the sample size and vice versa. Wide-mouth vials are preferable as they expand the maximum size of the specimens.
10. Polypropylene conical 50-ml tubes (with caps) for preparing the epoxy resin and lead citrate (e.g., 91050, Techno Plastic Products).
11. Long stainless-steel forceps to transfer the tissue and epoxy resin blocks between the solutions and short stainless-steel forceps (fine tip, curved) for specimen embedding (e.g., 12 and 4.5 inches, Z225622 and Z168785, Sigma-Aldrich).
12. Flexible stainless-steel wire ≈1–2 mm in diameter for removing the samples from the glass vials.
13. Single-channel, variable volume (20–200 μl and 100–1,000 μl) pipettes (e.g., 3123000055 and 3123000063, Eppendorf; Hamburg, Germany).
14. Disposable, clear 200–300- and 1,000-μl pipette philtre tips (e.g., TF-350 and TF-1000, Corning). May be non-sterile.
15. Gauze sponges for humidifying glass Petri dishes during lead citrate counterstaining (e.g., 8065-2, Covidien; Dublin, Ireland). May be non-sterile.

### Equipment

1. Double-distilled water system (e.g., A4000, Antylia Scientific) or deionised water system (e.g., Milli-DI Water Purification System for Deionized Water, Merck; Kenilworth, NJ, USA).
2. pH metre (e.g., pH 211, Hanna Instruments; Woonsocket, RI, USA).
3. Thermostat capable of heating to 60°C (70°C if Spurr low-viscosity resin is applied).
4. Vacuum impregnation unit (e.g., CitoVac, Struers).
5. Grinding and polishing machine (e.g., TegraPol-11, Struers).
6. Vacuum sputter coater (e.g., EM ACE200, Leica).
7. Scanning electron microscope equipped with a backscattered electron detector (e.g., Hitachi S-3400N, Hitachi; Tokyo, Japan) and, optionally, with an energy-dispersive x-ray detector (e.g., XFlash 4010, Bruker; Billerica, MA, USA).

## Methods

All steps of the procedure should be performed at RT unless otherwise stated.

**Figure 1:**
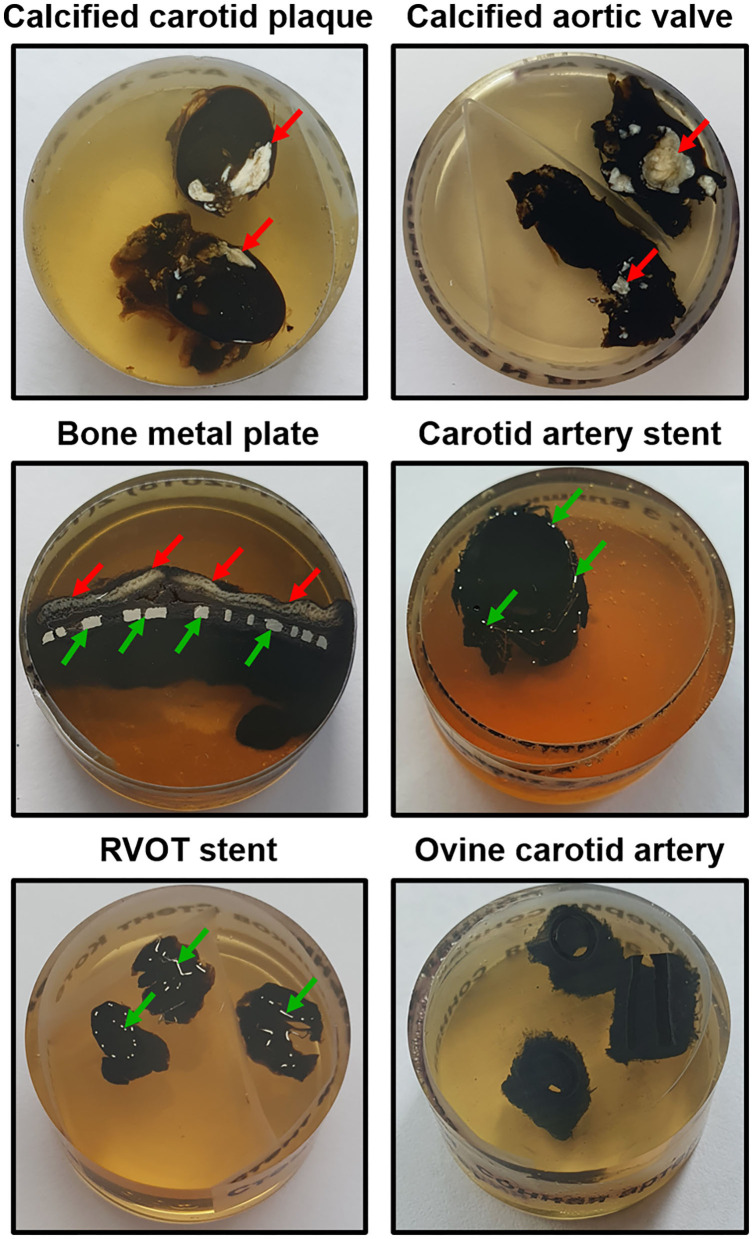
Appearance of grinded and polished epoxy resin blocks containing various tissue samples (mineralised carotid plaque, calcified aortic valve, bone metal plate, stented carotid artery and right ventricular outflow tract, and intact non-calcified ovine carotid artery). Calcified and metal inclusions are indicated by red and green arrows, respectively. RVOT, right ventricular outflow tract.

1. **DAY 1:** Carefully excise the tissue specimen and place it immediately into the transportation medium (ice-cold 1X phosphate-buffered saline or physiological saline). Ensure that the specimen is fully submerged into the transportation medium to prevent dehydration. Transfer the specimen into the tissue processing laboratory as soon as possible to prevent autolysis.
  **CRITICAL STEP:** The transportation medium (1X phosphate-buffered saline or physiological saline) and fixative (10% neutral phosphate-buffered formalin) must be ice-cold (4°C) to restrict autolysis as much as possible. Do not ever allow the tissue to dry out. At any step of the protocol, ensure that the specimen is fully submerged into the liquid.
2. Dissect the tissue specimen to ~5- ×5- ×5- (not more than 20- ×20- ×13-, L × W × H) mm segments if working with parenchymal organs. The advantage of cardiovascular tissue is that hollow organs such as blood vessels and thin heart valves are highly permeable for the formaldehyde molecules. Blood vessels and atherosclerotic plaques should be cut into ≈1-cm (not more than 1.3 cm) length segments for the convenience of embedding. The indicated dimensions ensure the proper and convenient orientation for epoxy resin embedding. The calcium deposits are typically well distinguishable from the surrounding tissue. To minimise the volume of the reagents used, calcified segments should be withdrawn just before formalin fixation. Wash the specimen in two or three changes of ice-cold 1X phosphate-buffered saline or physiological saline. Alternatively, intensively irrigate the specimen using a syringe if the tissue does not require a delicate treatment, as this technique is superior to dipping with regards to removing excessive blood.
3. Transfer the specimen into ice-cold 10% (vol/vol) neutral phosphate-buffered formalin as soon as possible to prevent autolysis. Despite the slower fixation rate, 10% neutral phosphate-buffered formalin is preferential to 4% paraformaldehyde and glutaraldehyde because of the smaller size and molecular weight of the molecules and, therefore, its better capability to penetrate the unprocessed tissue (32). Leave the sample overnight at 4°C.
  **PAUSE POINT:** The specimen can be safely stored at 4°C for up to a week without any quality loss.
4. **DAY 2:** Change the formalin in the morning and leave it until the end of a 24-h incubation period.
  **PAUSE POINT:** The specimen can be safely stored at 4°C for up to a week without any quality loss.
5. Wash the specimen by incubating it in 0.1 M phosphate buffer (pH 7.4, three changes, 10 min per each).
6. Transfer the specimen directly into a 1% phosphate-buffered osmium tetroxide solution. Hereinafter, strictly use glass and not plastic vials when working with osmium tetroxide to avoid any undesirable chemical interactions. Tightly wrap the container into a sealing film and leave the sample overnight at RT.
7. **DAY 3:** Transfer the specimen directly into a 2% aqueous osmium tetroxide solution. Tightly wrap the container into a sealing film and leave the sample for 40 h at RT.
  **PAUSE POINT:** The specimen can be safely stored at RT for a weekend (72 h) without any quality loss.
8. **DAY 5:** Wash the specimen by incubating it in 0.1 M phosphate buffer (pH 7.4, four changes, 15 min per each). Select the counterstain (UranyLess or uranyl acetate). If choosing UranyLess, proceed to a step 9, exclude steps 11 and 12, and continue the protocol from a step 13. If choosing uranyl acetate, skip step 9 and continue the protocol from step 10 (in this variant of the protocol, steps 10 and 11 would be performed at day 5 and day 6 would start at a step 12).
9. If using UranyLess for counterstaining, transfer the specimen directly into undiluted UranyLess and leave the sample overnight at RT.
  **CRITICAL STEP:** If planning an elemental analysis, UranyLess is strongly preferable to uranyl acetate, as lanthanides are typically not detected by energy-dispersive x-ray detector and, therefore, UranyLess staining does not result in an artefact peak, in contrast to uranyl acetate.
10. **DAY 6:** Transfer the specimen sequentially into 50, 60, 70, 80, and 95% ethanol (two changes per concentration, 15 min per change) without any washing steps.
11. If using uranyl acetate for counterstaining, transfer the specimen directly into freshly prepared 2% alcoholic uranyl acetate. Tightly wrap the container into a sealing film (if using uranyl acetate) and leave the sample overnight at RT. Protect from light.
12. Wash the specimen by incubating it in 95% ethanol (two changes, 15 min each).
13. Transfer the specimen directly into 2-propanol and incubate for 2 h. If the sample contains a high amount of fat, repeat the incubation in another change of 2-propanol.
14. Transfer the specimen directly into acetone and incubate for 2 h. If the sample contains a high amount of fat, repeat the incubation in another two changes of acetone (1 h per each).
15. Prepare the epoxy resin of desired formulation by (1) gentle mixing epoxy resin with a hardener/s until a homogeneous blend is obtained and (2) adding an accelerator and gently stirring until the mixture is uniform in colour.
  **CRITICAL STEP:** After the preparation, place an epoxy resin under vacuum to remove air bubbles as they might impede the embedding.
16. Transfer the specimen directly into a freshly prepared epoxy resin:acetone mixture (1:1) and leave overnight at RT.
17. **DAY 7:** Repeat step 15. Transfer the specimen directly into freshly prepared epoxy resin and leave overnight at RT.
18. **DAY 8:** Repeat the step 15.
  **CRITICAL STEP:** Upon preparation, it is mandatory to place the epoxy resin under vacuum to remove air bubbles as they might disrupt sample integrity during epoxy resin polymerisation.
19. Lubricate the polypropylene mounting cup from inside with silicone oil. Transfer the specimen directly into the polypropylene mounting cup and slowly fill the mould to a quarter with freshly prepared epoxy resin. Orientate the sample (e.g., blood vessels) if needed and continue filling the mould with epoxy resin until reaching the top of the cup. Place the mould into a thermostat and leave it for 24 h at 60°C.
  **CRITICAL STEP:** Ensure the proper orientation of samples at all times until placing the mounting cups into the thermostat. The orientation of the sample is unalterable upon the epoxy resin polymerisation, and improper orientation (e.g., falling of blood vessels) often leads to a loss of the sample.
  **CRITICAL STEP:** If working with hollow organs such as blood vessels, place the mould with the sample under vacuum to remove air bubbles before putting it into a thermostat.
20. **DAY 9:** Retrieve the epoxy resin blocks from the moulds. Sequentially grind the blocks until the sample surface is reached using the silicon carbide foil for the wet grinding of materials.
21. Polish the sample utilising wool-woven cloth discs and three monocrystalline diamond sprays (9-, 6-, and 3-μm diamond size, 20 min each) to flatten the surface of the epoxy resin block. The last stage of the polishing must be carried out without a diamond spray, i.e., using only a cloth disc for 20 min. Figure 1 illustrates the appearance of grinded and polished epoxy resin blocks containing various tissue samples.
  **CRITICAL STEP:** The use of a cooling and lubricating liquid is mandatory for high-quality diamond polishing.
22. Place the epoxy resin block onto wet gauze in a glass Petri dish. Counterstain the specimen by pipetting a lead citrate solution onto the surface of the epoxy resin block for 7 min. Ensure that the lead citrate fully covers the specimen surface.
  **CRITICAL STEP:** Prepare lead citrate solution at least 3 days before this step (i.e., not later than on day 6) to achieve better staining results.
  **CRITICAL STEP:** Close the Petri dish as soon as possible to prevent the contact of the lead citrate with air, which induces its precipitation.
  **CRITICAL STEP:** Do not counterstain the specimen with lead citrate if planning an elemental analysis as it will result in an artefact peak.
23. Wash the specimen by incubating the epoxy resin block in double-distilled or deionised water (three changes, 5 min each).
24. Sputter coat the epoxy resin block with carbon utilising a vacuum coater. An optimal sputtered coating thickness is 10–12 nm. If the sputter coating thickness exceeds 15 nm, repeat steps 21–24.
  **CRITICAL STEP:** At this step, do not touch an epoxy resin block without gloves as it may leave the fingerprints on the specimen. If this occasionally occurred, repeat steps 21–24.
  **CRITICAL STEP:** Set a carbon correction if planning an elemental analysis to exclude the contribution of carbon sputter coating to the elemental profile.
25. Visualise the specimen by means of BSEM at a 10- or 15-kV accelerating voltage. If using a Hitachi S-3400N electron microscope, set a BSECOMP mode. Perform the elemental analysis if desired employing an energy-dispersive x-ray detector.

### Timing

1. Fixation (10% neutral phosphate-buffered formalin): 24 h
2. Washing (0.1 M phosphate buffer): 30 min
3. Post-fixation (1% phosphate-buffered osmium tetroxide): 16 h
4. Staining (2% aqueous osmium tetroxide): 40 h
5. Washing (0.1 M phosphate buffer): 1 h
6. Counterstaining with UranyLess or 2% uranyl acetate: 16 h
7. Mild dehydration: 2.5 h
8. Degreasing/dehydration: 4 h (8 h if working with samples containing high amounts of fat)
9. Epoxy resin impregnation/degreasing/dehydration: 16 h
10. Epoxy resin impregnation: 24 h
11. Epoxy resin embedding (polymerisation): 24 h
12. Grinding and polishing: 2.5 h
13. Counterstaining with lead citrate: 7 min
14. Washing: 15 min
15. Sputter coating: 15 min
16. Visualisation and analysis: purpose-dependent

**Figure 2:**
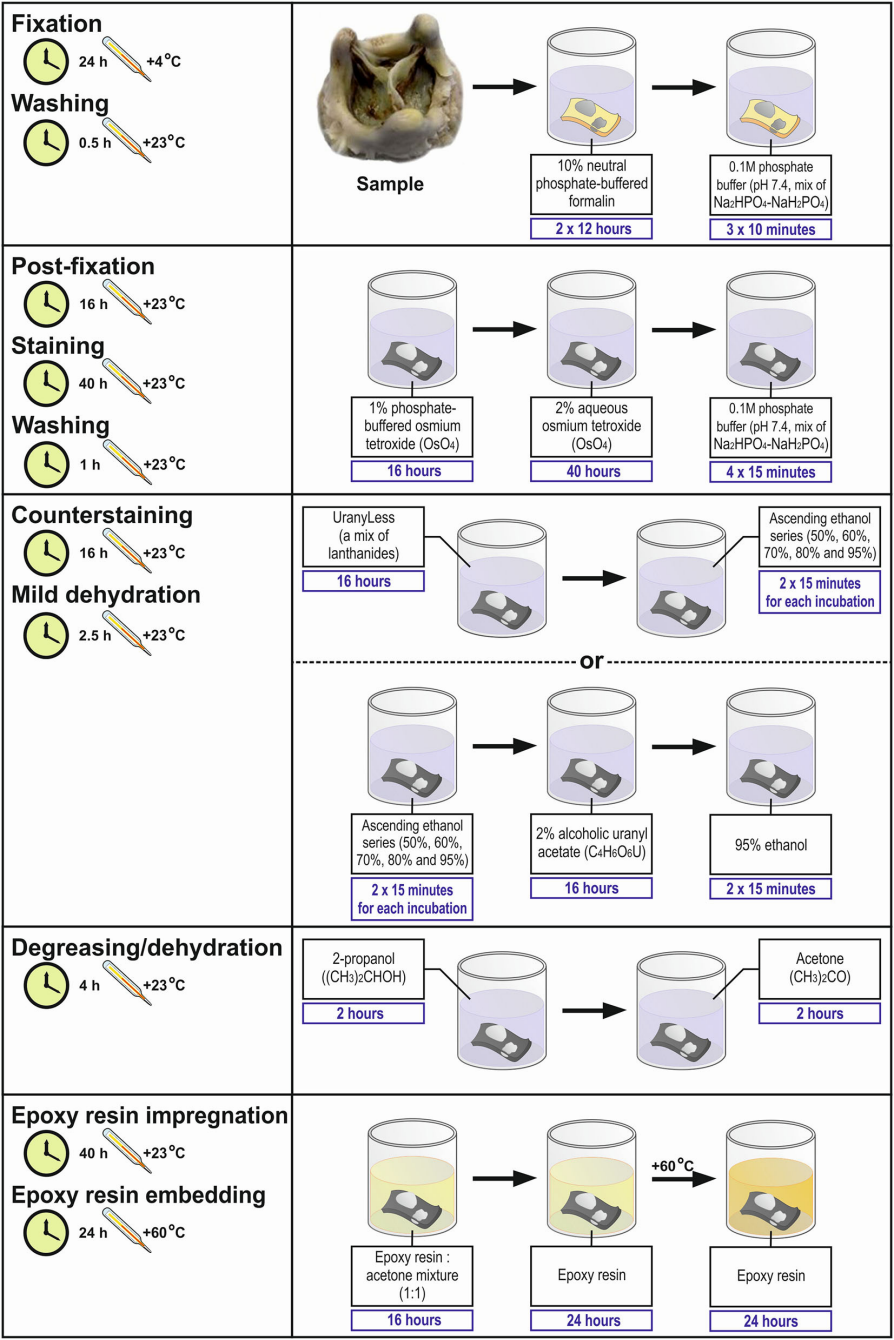
Workflow diagram for Embedding and Backscattered Scanning Electron Microscopy (EM-BSEM) from fixation to epoxy resin embedding.

**Figure 3:**
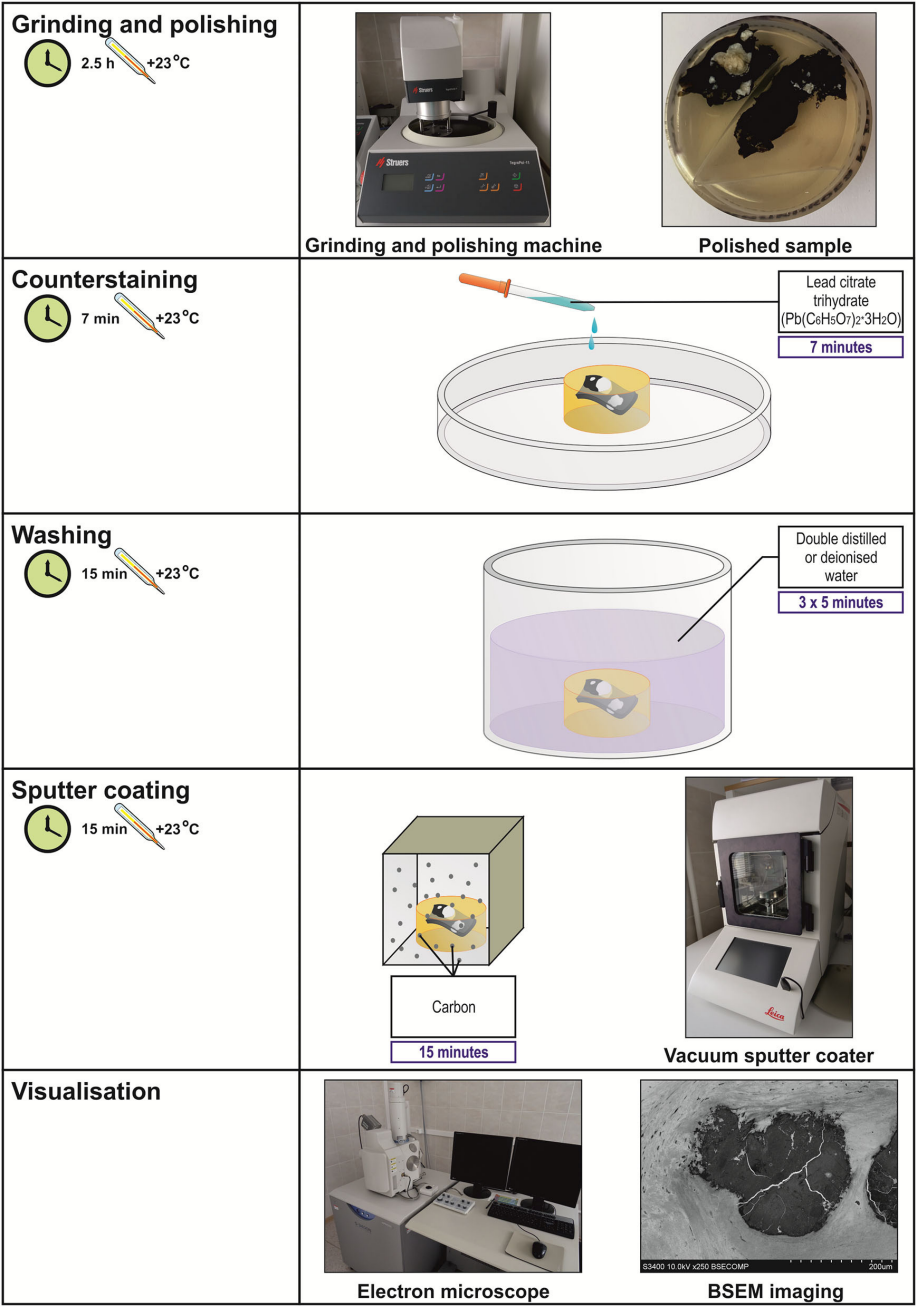
Workflow diagram for EM-BSEM from grinding and polishing to visualisation.

Total time: 171 h (Table 1, Figures 2, 3).

**Table 1:** Sequence and timing of the sample incubation in chemical reagents.

**Solution**	**Temperature**	**Time**
10% neutral phosphate buffered formalin	+4°C	2 ×12 h
0.1 M phosphate buffer (pH 7.4, mix of Na_2_HPO_4_-Na_2_HPO_4_)	RT	3 ×10 min
1% phosphate-buffered osmium tetroxide (OsO_4_)	RT	Overnight
2% aqueous osmium tetroxide (OsO_4_)	RT	40 h
0.1 M phosphate buffer (pH 7.4, mix of Na_2_HPO_4_-Na_2_HPO_4_)	RT	4 ×15 min
UranyLess (*if choosing lanthanide counterstaining*)	RT	Overnight
50% ethanol (CH_3_CH_2_OH)	RT	2 ×15 min
60% ethanol (CH_3_CH_2_OH)	RT	2 ×15 min
70% ethanol (CH_3_CH_2_OH)	RT	2 ×15 min
80% ethanol (CH_3_CH_2_OH)	RT	2 ×15 min
95% ethanol (CH_3_CH_2_OH)	RT	2 ×15 min
2% alcoholic uranyl acetate (C_4_H_6_O_6_U) (*if choosing uranyl acetate counterstaining*)	RT	Overnight
95% ethanol (*if choosing uranyl acetate counterstaining*)	RT	2 ×15 min
2-propanol [(CH_3_)_2_CHOH]	RT	2 h or 2 ×2 h
Acetone	RT	2 h or 2 + 2 ×1 h
Epoxy resin: acetone mixture (1:1)	RT	Overnight
Epoxy resin	RT	Overnight
Epoxy resin	+60°C	24 h
Lead citrate trihydrate [Pb(C_6_H_5_O_7_)_2_·3H_2_O]	RT	7 min
Double distilled or deionised water	RT	3 ×5 min

### Troubleshooting

1. If one adheres to the protocol, steps 1–18 generally do not cause any troubles. Regularly cheque the pH of the formalin solution. Allow the uranyl acetate to settle at least for 3 h upon its preparation. Always protect the uranyl acetate from air and light to avoid any precipitation. Gently mix the epoxy resin and a hardener, this blend and an accelerator, and ready-to-use epoxy resin and acetone until the mixtures become uniform in colour. Avoid any bubbles within the epoxy resin by a brief vacuuming to ensure proper impregnation and safe embedding. Control the storage temperature of all reagents and incubation temperature at all steps of the protocol.
2. At step 19 (embedding), if the sample lost its proper orientation (e.g., a blood vessel fell on its side), it can be liberated by employing a mini circular saw and embedded again.
3. Steps 20 and 21 are typically trouble-free if there is a sufficient amount of tap water and lubricating liquid during grinding and polishing, respectively.
4. Before conducting the step 22, cheque the pH and turbidity of the lead citrate solution. If the pH is below 11.9 or above 12.1, or if any precipitate is visible, prepare a fresh lead citrate solution. The precipitation of the lead citrate significantly contaminates the sample. If this occurs, prepare a fresh lead citrate solution and repeat steps 21–25. Always use freshly prepared double-distilled or deionised water while preparing the lead citrate to avoid its precipitation as it is highly reactive with carbon dioxide. In general, minimise the contact of the lead citrate with air.
5. Always cheque the thickness of the sputter coating as it must be from 10 to 15 nm to obtain a high-quality image.
6. If any scratches are observed during the visualisation, replace the polishing cloth disc and repeat steps 21–25.
7. If uranium and lead peaks are observed during the elemental analysis, replace uranyl acetate with UranyLess and do not perform lead citrate counterstaining. This will require repeating the procedure with the tissue backup.

## Anticipated Results

### Ethics Statement

All animal specimens (Table 2) have been collected in accordance with the European Convention for the Protection of Vertebrate Animals used for Experimental and Other Scientific Purposes (Strasbourg, 1986). The collection of human specimens (Table 3) conformed to the latest revision of the Declaration of Helsinki (2013). Written informed consent has been obtained from all individuals and medical care has been provided in accordance with the principles of Good Clinical Practise. The collection of all animal and human specimens has been approved by the Local Ethical Committee of the Research Institute for Complex Issues of Cardiovascular Diseases (Kemerovo, Russian Federation, protocol numbers AK_2019_02-10 and AK_2020_01-17 for the collection of animal and human specimens, respectively).

**Table 2:** Animal samples collected to suggest the usefulness of EM-BSEM in general and cardiovascular pathology.

**Animal**	**Excised tissue**	**Initial surgical intervention**	**Figures**
Male Wistar rat 6-months-old 500 g body weight	Descending aorta Heart Liver Spleen Kidney	None	Figures 4, 6
Male New Zealand White rabbit			
8-months-old	Skull	Cranioplasty	Figure 1
4.5 kg weight			
Female Edilbay sheep	Tissue-engineered carotid artery	Carotid artery interposition	
3-years-old 45 kg body weight	interposition graft	graft implantation for 1.5 years	Figures 1, 5

**Table 3:** Clinical samples collected to exhibit the applicability of EM-BSEM in cardiovascular pathology.

**Clinical sample**	**Patient**	**Cardiovascular pathology**	**Surgical intervention**	**Figures**
Atherosclerotic plaques	Female 58 years	Carotid atherosclerosis	Carotid endarterectomy	Figures 1, 5, 6, 8, 10
	Male 67 years	In-stent restenosis		Figures 1, 5
Calcified aortic valve	Male 66 years	Calcific aortic valve disease	Surgical aortic valve replacement	Figures 1, 5
Bioprosthetic xenopericardial bovine aortic valves	Female 72 years	Structural valve deterioration	Repeated surgical aortic valve replacement	Figures 5, 8
	Male 70 years			
	Female			
	72 years			Figure 8
Stented right ventricular outflow tract	Male 6 months	Tetralogy of Fallot	Total surgical repair	Figures 1, 5
Left internal mammary artery	Male 53 years	Chronic coronary syndrome	Pedicled internal mammary artery harvesting for coronary artery bypass graft surgery	Figures 6, 8, 9
Saphenous vein	Male 65 years	Chronic coronary syndrome	Open saphenous vein harvesting for coronary artery bypass graft surgery	Figure 7

### Applications of the Method

**Figure 4:**
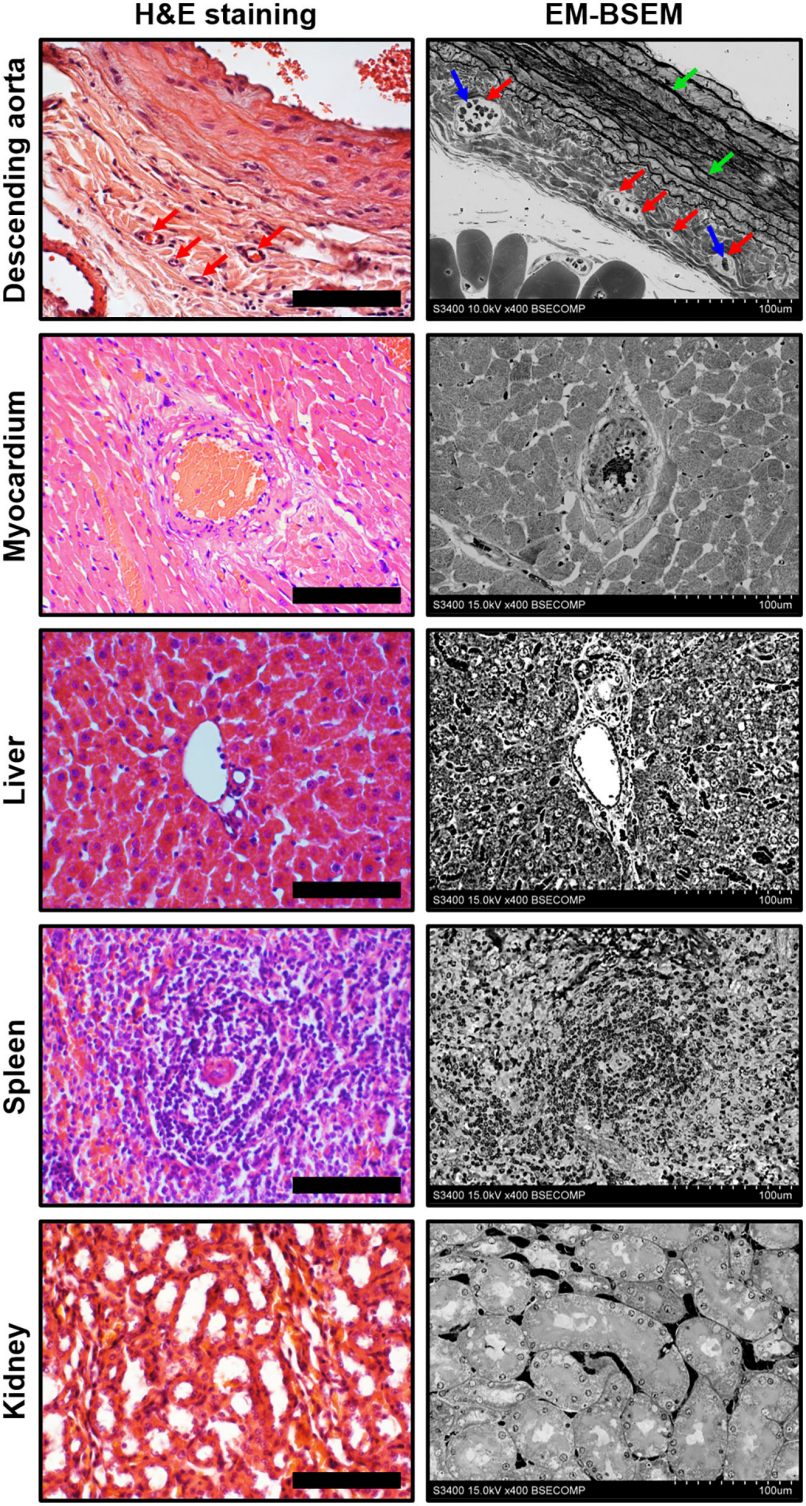
Side-by-side comparison of H&E staining and EM-BSEM of rat descending aorta, myocardium, liver, spleen, and kidney. Scale bar (100 μm) and magnification (×400) for H&E staining (left side) are equal to those on the BSEM images (right side). Accelerating voltage was 15 kV for all BSEM images, except that of the descending aorta (10 kV). The *vasa vasorum* in the tunica adventitia of the descending aorta are indicated by red arrows, while the red blood cells inside their lumen and elastic fibres are indicated by blue and green arrows, respectively.

Figure 4 illustrates the pairwise comparison of H&E staining, the most frequently applied pathological technique, and EM-BSEM at the highest magnification employed for scanning images for digital pathology needs (×400). When applied to blood vessels (e.g., rat descending aorta), EM-BSEM is preferable for annotating microvasculature in tunica adventitia and perivascular adipose tissue, as it clearly delineates blood vessel geometry and highlights red blood cells (Figure 4). Furthermore, it contrasts elastic fibres stained as black curves, which are easily distinguishable from the background, while that is not always the case for H&E staining (Figure 4). Another advantage of EM-BSEM is that it better preserves tissue integrity, excluding sectioning artefacts (Figure 4) and simplifying the preparation of specimens with a heterogeneous density, even those without calcium deposits. Although EM-BSEM has been initially designed for cardiovascular tissue, in particular calcified blood vessels, heart valves, and their bioprostheses, we suggest it can be applied for virtually all biological samples and for different research purposes (Figure 4).

We further demonstrate EM-BSEM applications in relation to the clinical samples of cardiovascular tissue (Table 3).

**Figure 5:**
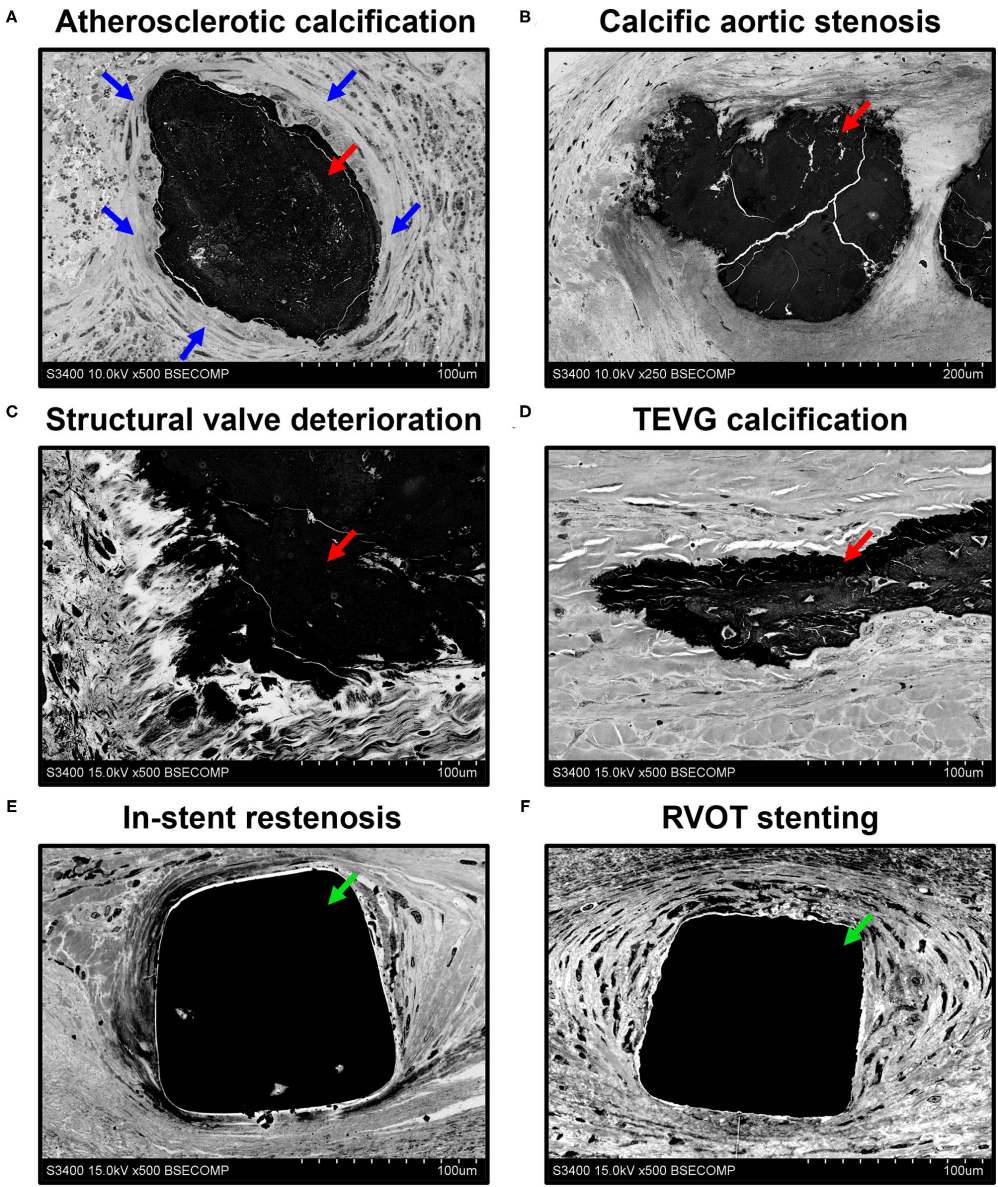
Visualisation of mineral deposits (indicated by red arrows) and metal implants (indicated by green arrows) in cardiovascular tissue by EM-BSEM shows the retained integrity of calcified [carotid plaque **(A)**, native **(B)**, and bioprosthetic **(C)** aortic valves, and tissue-engineered vascular grafts implanted into the ovine carotid artery **(D)**] and metal-incorporating tissues [human carotid artery **(E)** and right ventricular outflow tract **(F)**]. Note the clear-cut connective tissue capsule (indicated by blue arrows) around the atherosclerotic but not the valvular or vascular graft calcifications. A magnification of ×500 (scale bar: 100 μm) for all images, except that of the calcified native aortic valve (×250 magnification, scale bar: 200 μm). The accelerating voltage was 15 kV for all images, except those of the atherosclerotic plaque and calcified native aortic valve (10 kV).

The assessment of calcifications and stents in different parts of the circulatory system revealed the fully retained integrity of the surrounding tissue regardless of location or pathological condition (Figure 5). Atherosclerotic calcifications were frequently demarcated by a connective tissue capsule (Figure 5A) in contradistinction to valvular mineral deposits, which developed during calcific aortic stenosis (Figure 5B) or the structural deterioration of bioprostheses within the disorganised extracellular matrix (Figure 5C). Calcifications on the border between the tunica media and tunica adventitia within the tissue-engineered vascular grafts (Figure 5D) resembled medial arterial calcification, which is a common finding in patients with advanced chronic kidney disease and end-stage renal disease (33, 34). The connective tissue enveloping metal stents upon carotid angioplasty (Figure 5E) or right ventricular outflow tract stenting (Figure 5F) was also intact, permitting an analysis of biocompatibility of these devices.

**Figure 6:**
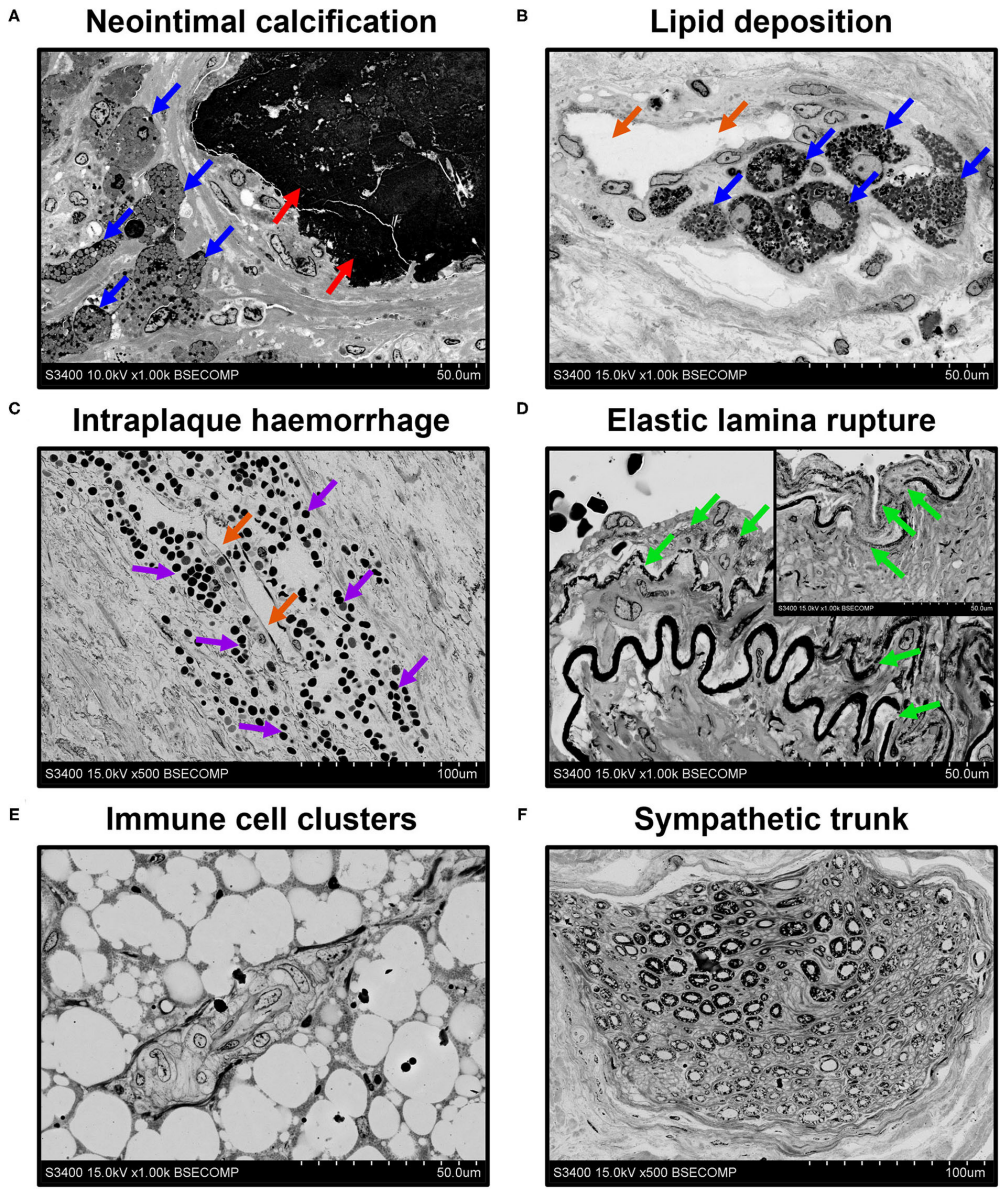
EM-BSEM imaging of cardiovascular pathology. Note the abundance of **(A,B)** foam cells (indicated by blue arrows) and **(C)** red blood cells (indicated by violet arrows) near the calcium deposit (indicated by red arrows) and neovessels (indicated by orange arrows) within the carotid plaque. **(D)** Also note the age-dependent degradation of the elastic laminae (indicated by green arrows) of the left internal mammary artery excised for coronary artery bypass graft surgery purposes. Note the vascular smooth muscle cells between the elastic laminae and the traceable contours of the internal elastic lamina repeating that of the following elastic laminae. The insert in the upper-right corner indicates the visible decomposition of two sequential elastic laminae. **(E)** Immune cell clusters frequent in the adventitia and perivascular adipose tissue of rat descending aorta undergo hyperplasia at inflammation and might represent a valuable marker of immune response but should be clearly distinguished from **(F)** a sympathetic trunk. A magnification ×1,000 (scale bar: 50 μm) for all images, except, that of intraplaque haemorrhage and sympathetic trunk (x500 magnification, scale bar: 100 μm). The accelerating voltage was 15 kV for all images excepting that of neointimal calcification (10 kV).

An important benefit of EM-BSEM is that it allows the detailed evaluation of blood vessels and heart valves, as most of cardiovascular pathology phenomena can be investigated at ≤ ×5,000 magnification. As portrayed in Figure 6, EM-BSEM can be applied to study neointimal calcification (Figure 6A), which is often accompanied by the alterations of cellular phenotype such as the transformation of macrophages or vascular smooth muscle cells into foam cells because of the massive lipid deposition near the vessel lumen or around *vasa plaquorum* (Figure 6B) (35–38). The progression of atherosclerosis is also enhanced by intraplaque haemorrhages occurring through leaky plaque neovessels (36, 39–41) and is notable for the large amount of red blood cells in their vicinity (Figure 6C). A mandatory condition of neointimal hyperplasia, however, is breach of the internal elastic lamina (42, 43), which is well detectable by EM-BSEM and can even be quantified (Figure 6D). The development of neointima is associated with increased quantities of immune cell clusters within the adventitia and perivascular adipose tissue (Figure 6E), in agreement with an outside–in route of atherosclerosis progression (44–47). Furthermore, EM-BSEM affords both the high-quality visualisation of these structures and their distinction from the sympathetic trunk (Figure 6F).

**Figure 7:**
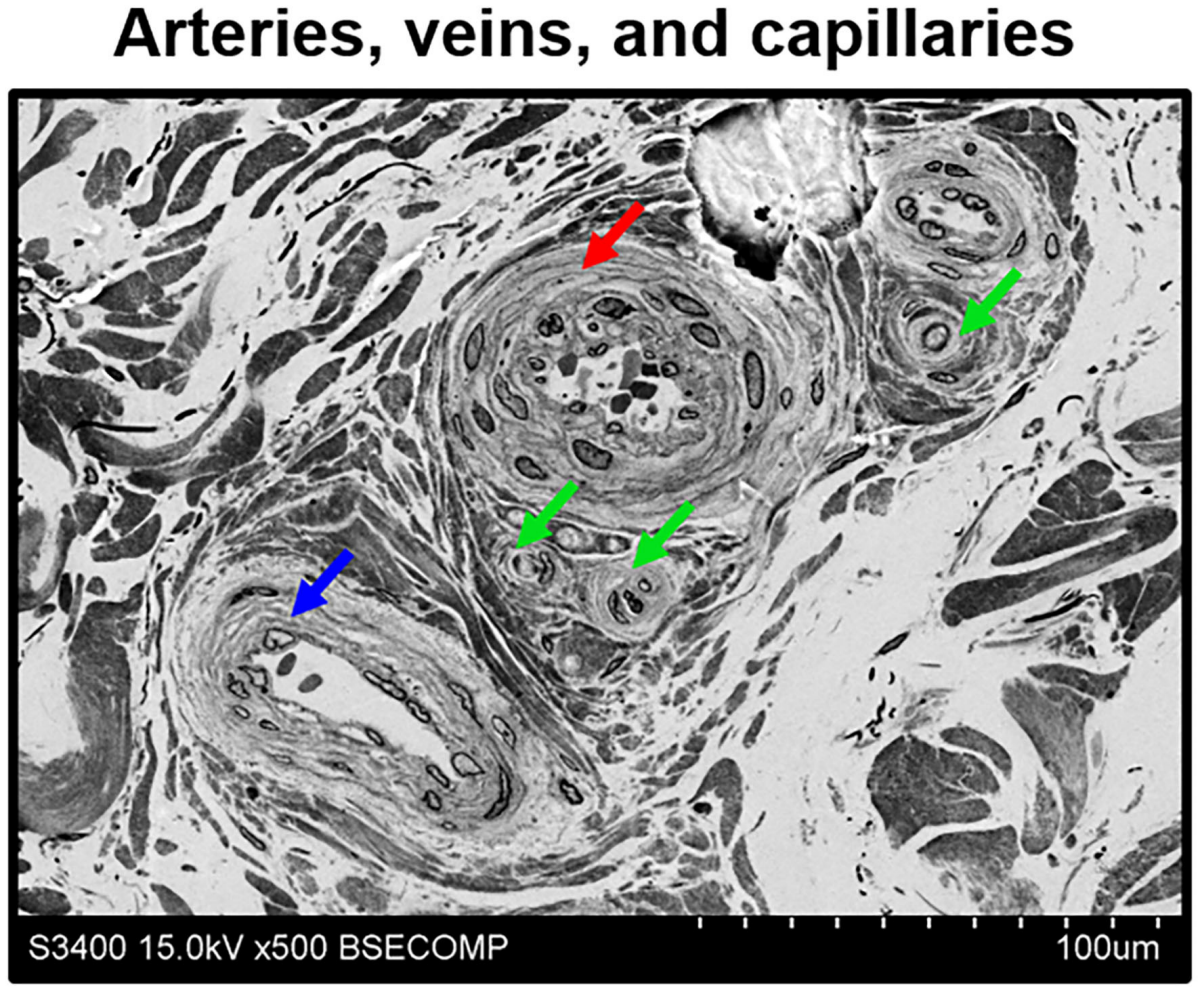
Discrimination of adventitial arterioles, venules, and capillaries by means of EM-BSEM. Human saphenous vein excised for coronary artery bypass graft surgery purposes. Note that despite all microvessels having an endothelial cell monolayer, arterioles (indicated by a red arrow) and venules (indicated by a blue arrow) additionally include ≥1 concentric layer of vascular smooth muscle cells, in contrast to capillaries (indicated by green arrows). In general, arterioles have a narrow lumen and often have large smooth muscle cells, while venules are typically distended and their smooth muscle cells are smaller. A magnification of ×500 (scale bar: 100 μm), 15 kV accelerating voltage.

A compulsory requirement for any histological approach applicable in vascular biology is its ability to differentiate the arterioles nourishing the blood vessels, venules removing metabolic wastes, and capillaries responsible for the exchange of gases, nutrients, and waste products. As shown in Figure 7, EM-BSEM discriminates concentric arterioles consisting of endothelium and several smooth muscle cell layers, distended collecting venules, and tiny capillaries whose diameters are similar to that of a red blood cell. This indicates its potential usefulness to analysing the evolution of the microvascular networks in growing blood vessels, tissue-engineered constructs, and for other developmental biology applications.

**Figure 8:**
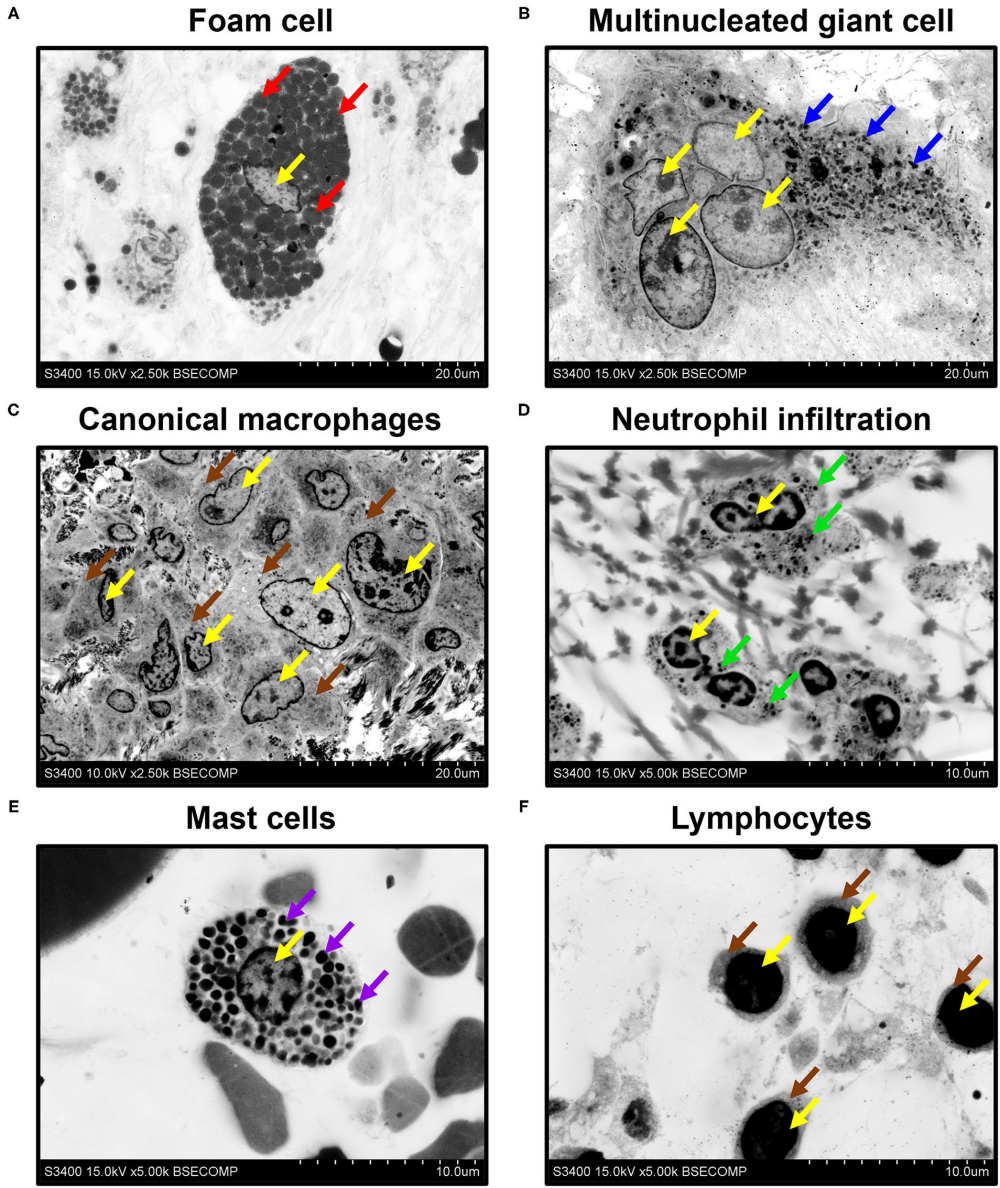
Phenotyping of immune cells and mast cells by EM-BSEM. Nuclei are indicated by yellow arrows. Note **(A)** the large spherical lipid granules within the foam cells of carotid plaque (indicated by red arrows) and **(B)** multiple small granules within the multinucleated (foreign body) giant cells of the bioprosthetic aortic valve (indicated by blue arrows). **(C)** Canonical macrophages of the same valve lack granules within the cytoplasm (indicated by brown arrows). **(D)** The neutrophils of rapid-failing bioprosthetic aortic valve were characterised by numerous small granules within the cytosol (indicated by green arrows) and polymorphic nuclei. **(E)** In contrast, mast cells from the perivascular (human coronary artery) adipose tissue have large spherical inclusions in the cytosol (indicated by violet arrows) and round nuclei. **(F)** Lymphocytes from the same adipose tissue sample were distinct from other cells because of a high nuclear-cytoplasmic ratio (cytoplasm is indicated by brown arrows): ×2,500 magnification (scale bar: 20 μm) for images of foam cells, multinucleated giant cells, and canonical macrophages; ×5,000 magnification (scale bar: 10 μm) for images of neutrophils, mast cells, and lymphocytes. The accelerating voltage was 15 kV for all images, except that of canonical macrophages (10 kV).

All mentioned applications, however, generally do not demand high (> ×1,000) magnifications, although a possibility to operate within the ×400–1,000 magnification range and to acquire high-resolution images is beneficial for the analysis of tissue architecture. Yet, higher magnifications are needed to investigate ultrastructural cardiovascular pathology and to identify the characteristic features of cell populations. Employing EM-BSEM, we were able to image foam cells with clear-cut lipid droplets in the cytosol (Figure 8A), multinucleated giant cells containing processed components of the extracellular matrix (Figure 8B), and canonical macrophages having round or oval nuclei (Figure 8C). For this reason, EM-BSEM can be particularly advantageous for the analysis of atherosclerotic plaques that contain major amounts of foam cells (i.e., macrophages and vascular smooth muscle cells engulfing lipid molecules) and bioprosthetic heart valves, which are notable for multiple foreign-body giant cells (11). In addition, EM-BSEM also discerns neutrophils, which have segmented nuclei and small granules (Figure 8D), mast cells with a round nucleus and large electron-dense granules (Figure 8E), and lymphocytes differing from other immune cells by a high nuclear-cytoplasmic ratio (Figure 8F).

**Figure 9:**
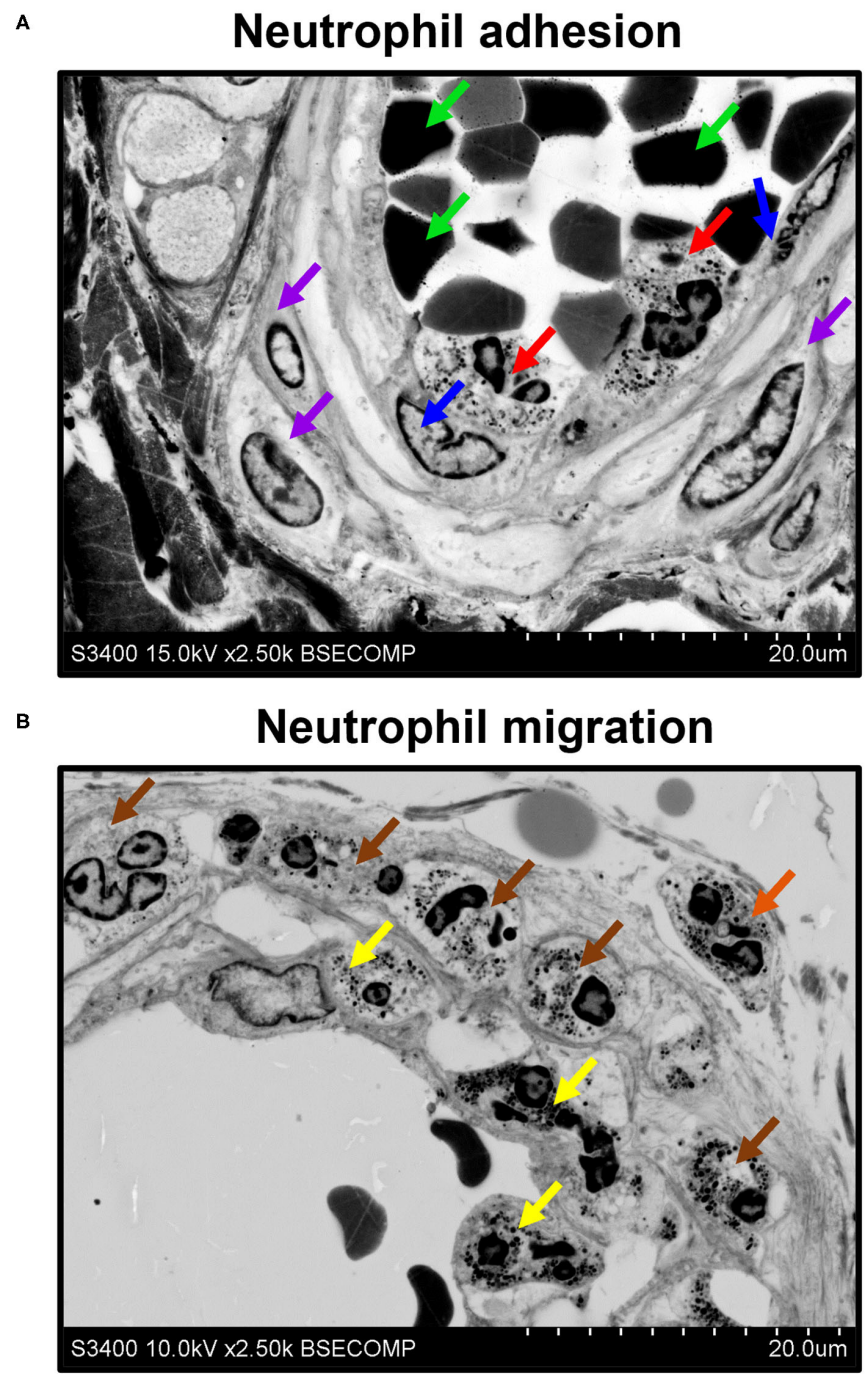
EM-BSEM visualisation of neutrophil adhesion and migration. Left internal mammary artery excised for coronary artery bypass graft surgery purposes. **(A)** Note the neutrophils (indicated by red arrows) adhering to endothelial cells (indicated by blue arrows). Red blood cells and vascular smooth muscle cells are indicated by green and violet arrows, respectively. **(B)** Note adhering neutrophils (indicated by yellow arrows), invading neutrophils (indicated by brown arrows), and migrating neutrophils (indicated by an orange arrow). A magnification of ×2,500 (scale bar: 20 μm) and accelerating voltage of 15 kV (upper image) and 10 kV (lower image).

Besides the descriptive findings, EM-BSEM might be harnessed to investigate pathophysiological events. For instance, we noticed the adhesion of neutrophils to endothelial cells (Figure 9A) and their migration through the vascular wall (Figure 9B) in the *vasa vasorum* locating at the perivascular adipose tissue of human internal mammary arteries, which are frequently used as conduits for coronary artery bypass graft surgery. The applicability of EM-BSEM to vascular pathophysiology is underscored by the opportunity to clearly distinguish endothelial cells, vascular smooth muscle cells (Figure 9), adventitial cell populations (macrophages, mast cells, and fibroblasts), and perivascular adipocytes (Supplementary Figure 1).

**Figure 10:**
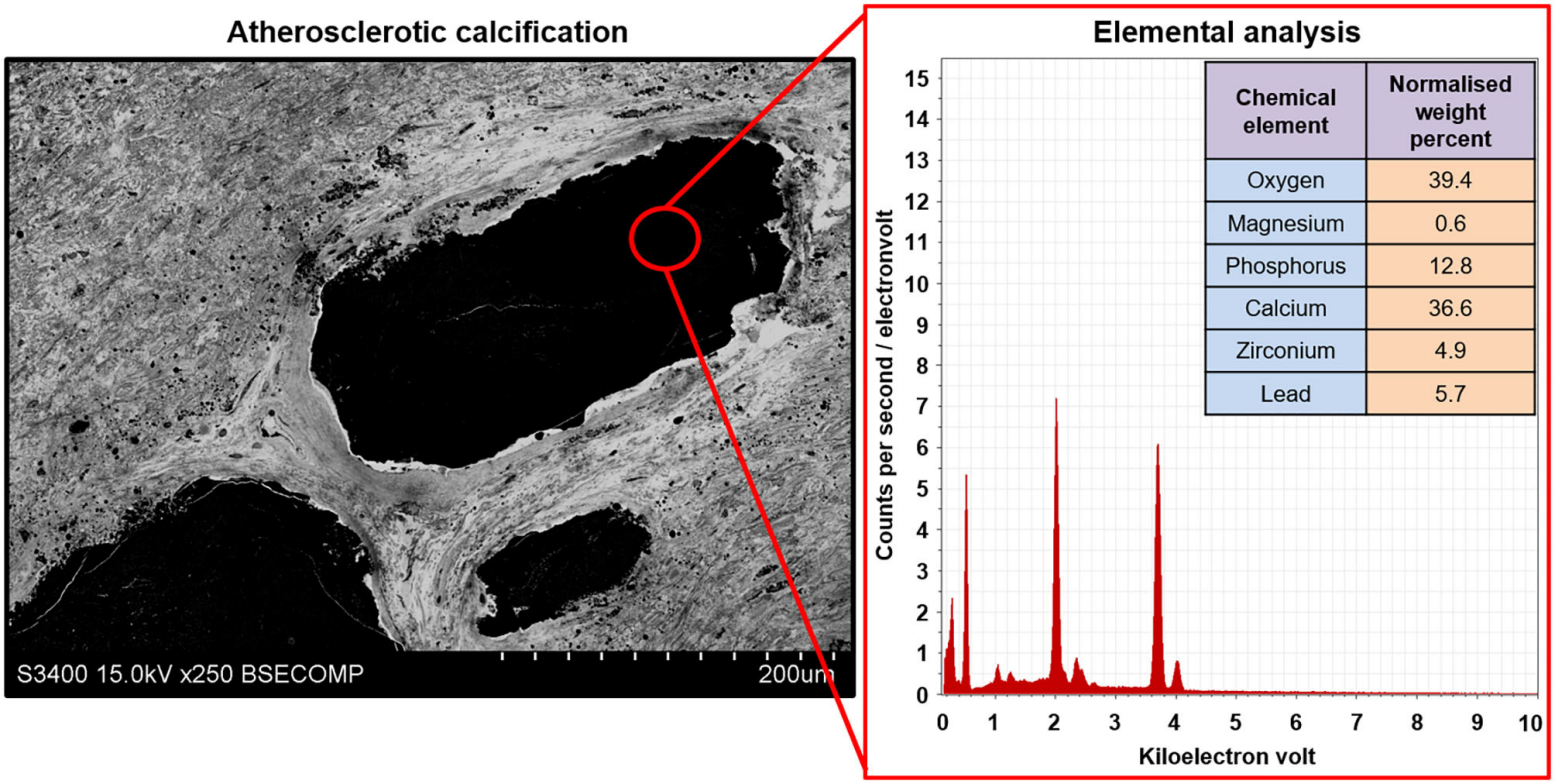
Elemental analysis of the atherosclerotic calcium deposit within the carotid plaque visualised employing EM-BSEM. Note the high calcium, phosphorus, and oxygen content suggestive of a hydroxyapatite. For a more precise elemental analysis, uranyl acetate should be replaced by UranyLess, and lead citrate should be avoided. Yet, this results in a worse detailing of cells and the extracellular matrix. A magnification of ×250 (scale bar: 200 μm) and accelerating voltage of 15 kV.

In addition to conventional cardiovascular biology applications, EM-BSEM can be coupled with an elemental analysis to interrogate the chemical composition and heterogeneity of calcifications (Figure 10).

## Discussion

To our knowledge, all currently available histological techniques include sectioning, which inflicts irreversible damage to calcified and stent-expanded tissues and are, therefore, unsuitable for the subsequent analysis of such specimens. Furthermore, none of the existing techniques allows the detailed and high-throughput analysis of cardiovascular microanatomy. Vascular and valvular cell types are often barely distinguishable even at the highest conventional LM magnification (400-fold) if routine stains are applied. In addition, a significant proportion of small-calibre *vasa vasorum* and adventitial or perivascular immune cell clusters cannot be well-discriminated from the surrounding connective tissue and quantified by means of LM.

Confocal and multiphoton microscopy permits 3D image acquisition with the subsequent visualisation, qualitative assessment, and quantification of cell populations, cellular structures, and cell–cell interactions (48–50). In contrast to single-photon confocal microscopy, multiphoton (e.g., two-photon) microscopy uses longer-wavelength photons that penetrate deeper into the tissue (500 μm−1 mm) and inflict less damage on the sample (48). The main advantage of two-photon microscopy is its better focus, allowing the obtainment of clearer images as compared to single-photon confocal microscopy (48). The combination of a high penetration capability and low phototoxicity provides an opportunity to visualise the undissected tissues of living animals (48, 50). However, two-photon microscopy requires expensive and highly specific equipment that significantly confines its use to specialised laboratories having certified specialists employed. Furthermore, immunostaining demarcates specific cell types or extracellular matrix components but is not informative of tissue architecture.

The mentioned drawbacks can be resolved by TEM, which is incompatible with large samples (e.g., most of the blood vessels) due to a critically reduced amount of analysable tissue, needs difficult sample preparation, and the final sample represents a single tissue snapshot rather than serial sections evenly distributed along or across the specimen. Notably, the visualisation of calcified tissues is often performed by means of density-dependent scanning electron microscopy (SEM), which implies the superimposing of images acquired by secondary and backscattered electron detectors and further assigning them to the distinct colour channels (e.g., green and red), thereby permitting the coloured mapping of calcific nodules and distinguishing them from soft tissues (51–53). The main advantage of this technique is that it allows the simultaneous visualisation of both topography and density in a single image (51). Although it is perfect for the visualisation and morphometrics of calcifications, density-dependent SEM generally does not furnish the ultrastructural details of their biological microenvironments and, therefore, the phenotyping of the neighbouring cell populations is complicated.

In biomedicine, BSEM is broadly used for the imaging of unstained, freeze-dried cell cultures (54), wet calcified atherosclerotic lesions (55), implant-bone interface (56), cartilage and bone tissue (57), and a diversity of other tissues (58). Despite high-resolution BSEM and field emission SEM having been earlier employed for the multiscale visualisation of semi-thin and ultra-thin sections, respectively (58, 59), these sample preparation approaches are inconvenient when working with mineralised tissues or specimens with incorporated solid implants. The techniques of correlative histology [i.e., a time-resolved combination of imaging modalities from lower to higher magnifications (1, 60)], such as the three-sectioning method, where epoxy resin-embedded tissue is sequentially cut into thick (≈300 μm), semi-thin (1–3 μm), and ultra-thin (60–90 nm) sections to specify regions of interest, have been proposed for working with hard tissues (61). However, correlative microscopy is typically time consuming, labour intensive, and is designed to focus on a few tissue segments rather than for screening purposes and semi-quantitative image analysis. Another correlative histology approach includes cryosectioning and combination of immunogold labelling with subsequent confocal laser scanning microscopy (62). Then, stained sections on the glass slides are fixed in glutaraldehyde, incubated in a gold-enhancing solution to enlarge nanogold particles, stained with osmium tetroxide, uranyl acetate, and lead citrate, and dehydrated and embedded into epoxy resin with the following ultramicrotome sectioning, with repeated adhering to a glass slide, carbon coating, and BSEM (62). Alternatively, specific acrylic resins (e.g., LR White) enable the post-embedding immunofluorescence staining and confocal laser scanning microscopy of semi-thin sections, which can then be stained with heavy metals and prepared for BSEM (63–65). This correlative microscopy approach has the advantage of combining the immunophenotyping and ultrastructural examination of the same field of view, though to the detriment of the area available for the analysis and being incompatible with calcified tissues. A recent protocol suggested by Goggin et al. (66) implies glutaraldehyde fixation, 1-week decalcification with ethylenediaminetetraacetic acid, and combined staining with 2% osmium tetroxide and 1.5% potassium ferrocyanide, with a subsequent incubation in thiocarbohydrazide [to cross-link the osmium layers and enhance further osmium binding by bridging two osmium molecules (67–69)], pure osmium tetroxide, uranyl acetate, and Walton's lead aspartate solutions followed by dehydration and resin embedding (66). Albeit it provides good results with regards to hard tissues (e.g., bone), prolonged exposure to ethylenediaminetetraacetic acid deteriorates tissue integrity and reduces image quality when visualising soft tissues, e.g., cardiovascular tissue surrounding calcium deposits. In addition to working with tissues, ultra-thin epoxy resin embedding has also been applied to the preparation of cell cultures for scanning electron microscopy to image individual cells and cell–cell interactions on planar and three-dimensional substrates in preference to critical point drying, but this technique was not optimised for BSEM to visualise intracellular compartments (70).

Importantly, high-resolution images have been obtained only in studies examining semi-thin and ultra-thin sections (58, 59), where staining with uranyl acetate and lead citrate enabled the reaching of a BSEM resolution comparable to that of transmission electron microscopy because such samples can be fully penetrated by an electron beam. However, this method demands tissue sectioning, which rarely allows one to keep the integrity of the samples including mineral deposits or metal implants. Our technique (EM-BSEM) entirely retains the integrity of calcified or metal-containing samples, affords the analysis of their chemical composition, and combines the advantages of LM and TEM, providing both gross and high-resolution images and detailed histological characteristics of all vascular and valvular tissue structures and cell types. The sequential grinding of the sample permits consecutive, layer-by-layer scanning that is principally similar to serial sectioning but does not cause any damage to mineralised tissues. The sample preparation is relatively straightforward and has only one extremely critical step (sample orientation during epoxy resin embedding) with a high risk of losing the sample. During visualisation, the penetration depth of an electron beam depends on accelerating voltage, beam current, and the concentration of heavy metals at or beneath the surface of the sample. The first two parameters are tuned while visualising the sample while the latter is optimised when designing an experimental pipeline. Here, we presented a detailed protocol that enables (1) the retaining of the integrity of calcified and stent-expanded cardiovascular tissues; (2) the obtainment of high-resolution images, thus allowing the visualisation of mineral deposits and their microenvironment, annotation of different microvessel types (i.e., arterioles, venules, and capillaries), and identification of various cardiovascular pathologies and cell populations; (3) the elemental analysis of calcifications. The protocol has been optimised for working with cardiovascular tissues but can be applied for virtually any tissue. A comparison of the features attributed to LM, confocal or multiphoton microscopy, TEM, density-dependent SEM, and EM-BSEM are represented in Table 4.

**Table 4:** Comparison of light microscopy (LM), EM-BSEM, and transmission electron microscopy (TEM).

**Feature**	**LM**	**Confocal/multiphoton microscopy**	**TEM**	**Density-dependent SEM**	**EM-BSEfM**
Resolution	Low	Low	High	High	Average
Representativeness	High	High	Low	High	High
Informativeness	Average	Average	Average	Average	High
Versatility	Average	Average	Average	Low	High
Technical complexity	Average	High	High	Low	Low
Labour intensity	Average	Average	High	Average	Average
Hands-on time	Average	Average	High	Average	Average
Time to complete	Average	Average	High	Average	High

*LM, light microscopy; TEM, transmission electron microscopy; SEM, scanning electron microscopy; EM-BSEM, EMbedding and Backscattered Scanning Electron Microscopy*.

The possible limitation of our protocol is that it is hardly compatible with immunoelectron microscopy because of its insufficient resolution [even the largest gold particles labelling the secondary antibodies (25 nm in diameter) are barely detectable even at ×10,000 magnification], although some acrylic resins (e.g., LR White) allow the penetration of primary and secondary antibodies into the embedded tissue.

We suggest that EM-BSEM is superior to both H&E staining and TEM for studying cardiovascular pathology. The benefits of EM-BSEM include: (1) an image resolution sufficient for both the gross and detailed examination of elastic lamina degradation, (neo)intimal hyperplasia, (neo)vascularisation, intraplaque or intravalvular haemorrhages, lipid retention and foam cell formation, and disintegration of the extracellular matrix (including those mediated by the specialised macrophages); (2) a reliable identification of vascular cell populations (endothelial cells, vascular smooth muscle cells, macrophages, fibroblasts, mast cells, and adipocytes) and the recognition of immune cell lineages (macrophages, foam cells, foreign-body giant cells, neutrophils, and lymphocytes); (3) an opportunity to perform a chemical analysis of mineral deposits and metal implants in combination with obtaining a holistic overview of their tissue environment; (4) a technical possibility to quantify any distinguishable feature across the entire sample surface (e.g., blood vessel area) and at variable depth through the serial grinding; (5) a compatibility with modern machine learning algorithms for the automated annotation of histological and cellular patterns; (6) low technical complexity together with moderate labour intensity and hands-on time. Although the sample preparation in EM-BSEM is possibly too long for the broad implementation of this approach to clinical medicine, we assume it can be widely established in basic and translational research and would probably find its applications in other branches of biomedical science beyond the cardiovascular field.

## Data Availability Statement

The original contributions presented in the study are included in the article/Supplementary Material, further inquiries can be directed to the corresponding author.

## Ethics Statement

The animal study was reviewed and approved by Local Ethical Committee of the Research Institute for Complex Issues of Cardiovascular Diseases. Studies involving human subjects were reviewed and approved by Local Ethical Committee of the Research Institute for Complex Issues of Cardiovascular Diseases. The patients/participants provided their written informed consent to participate in this study.

## Author Contributions

RM conceived and designed the EM-BSEM approach and prepared Tables 1, 4, Figures 5, 6, 8. LB, TG, and DS prepared Figures 4, 6, 7, 9, 10. AEK prepared Figures 2, 3. VK prepared Figure 1. ARS provided the clinical specimens for the preparation of Figures 1, 5, 6, 8, 10. AF provided the clinical specimens for the preparation of Figures 6–9. ANS provided the clinical specimens for the preparation of Figures 1, 5, 8. AL provided the clinical specimens for the preparation of Figures 1, 5. AGK prepared Tables 2, 3 and wrote the manuscript. All authors contributed to the article and approved the submitted version.

## Funding

This study was supported by the Complex Program of Basic Research under the Siberian Branch of the Russian Academy of Sciences within the Basic Research Topic of Research Institute for Complex Issues of Cardiovascular Diseases No. 0546-2019-0002. Pathogenetic basis for the development of cardiovascular implants from biocompatible materials using a patient-oriented approach, mathematical modelling, tissue engineering, and genomic predictors.

## Conflict of Interest

The authors declare that the research was conducted in the absence of any commercial or financial relationships that could be construed as a potential conflict of interest.

## Publisher's Note

All claims expressed in this article are solely those of the authors and do not necessarily represent those of their affiliated organizations, or those of the publisher, the editors and the reviewers. Any product that may be evaluated in this article, or claim that may be made by its manufacturer, is not guaranteed or endorsed by the publisher.
