# Supplementary material for: EMbedding and Backscattered Scanning Electron Microscopy: A Detailed Protocol for the Whole-Specimen, High-Resolution Analysis of Cardiovascular Tissues
Source: Front Cardiovasc Med. 2021 Oct 25;8:739549. doi: 10.3389/fcvm.2021.739549 (PMC8573413; doi:10.3389/fcvm.2021.739549)
Supplement: Supplementary file 1 [file Data_Sheet_1.docx]

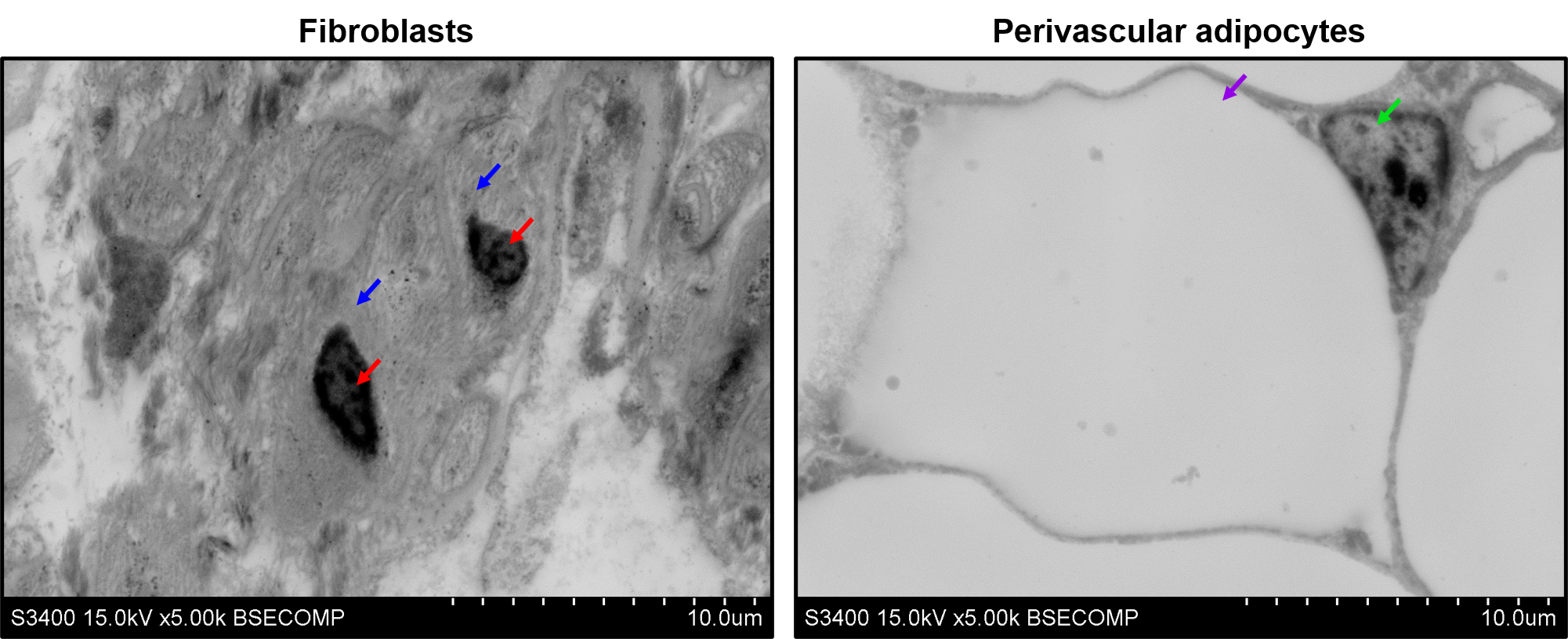


**Supplementary Figure 1. EM-BSEM identifies fibroblasts and perivascular adipocytes.** Note the polygonal nuclei (indicated by red arrows) and elongated cytoplasm (indicated by blue arrows) of fibroblasts as well as small nuclei (indicated by a green arrow) and delipidated cytoplasm (indicated by a violet arrow) of a perivascular adipocyte. x5,000 magnification (scale bar: 10 µm), 15 kV accelerating voltage for both images.
